# Xylose donor transport is critical for fungal virulence

**DOI:** 10.1371/journal.ppat.1006765

**Published:** 2018-01-18

**Authors:** Lucy X. Li, Carsten Rautengarten, Joshua L. Heazlewood, Tamara L. Doering

**Affiliations:** 1 Department of Molecular Microbiology, Washington University School of Medicine, St. Louis, Missouri, United States of America; 2 School of Biosciences, The University of Melbourne, Melbourne, VIC, Australia; University of Birmingham, UNITED KINGDOM

## Abstract

*Cryptococcus neoformans*, an AIDS-defining opportunistic pathogen, is the leading cause of fungal meningitis worldwide and is responsible for hundreds of thousands of deaths annually. Cryptococcal glycans are required for fungal survival in the host and for pathogenesis. Most glycans are made in the secretory pathway, although the activated precursors for their synthesis, nucleotide sugars, are made primarily in the cytosol. Nucleotide sugar transporters are membrane proteins that solve this topological problem, by exchanging nucleotide sugars for the corresponding nucleoside phosphates. The major virulence factor of *C*. *neoformans* is an anti-phagocytic polysaccharide capsule that is displayed on the cell surface; capsule polysaccharides are also shed from the cell and impede the host immune response. Xylose, a neutral monosaccharide that is absent from model yeast, is a significant capsule component. Here we show that Uxt1 and Uxt2 are both transporters specific for the xylose donor, UDP-xylose, although they exhibit distinct subcellular localization, expression patterns, and kinetic parameters. Both proteins also transport the galactofuranose donor, UDP-galactofuranose. We further show that Uxt1 and Uxt2 are required for xylose incorporation into capsule and protein; they are also necessary for *C*. *neoformans* to cause disease in mice, although surprisingly not for fungal viability in the context of infection. These findings provide a starting point for deciphering the substrate specificity of an important class of transporters, elucidate a synthetic pathway that may be productively targeted for therapy, and contribute to our understanding of fundamental glycobiology.

## Author summary

*Cryptococcus neoformans*, the leading cause of fungal meningitis, kills almost a quarter of a million people each year. Carbohydrate structures, including a complex polysaccharide capsule, enable this microbe to resist host defenses and cause disease. The machinery to build these structures is located inside of specific subcellular compartments. However, most of the reactions require as precursors activated sugar molecules that are made in the cell cytosol. To bring these compounds, called nucleotide sugars, to the site of synthesis, the cell expresses specific transporters. One precursor of particular interest is UDP-xylose, which is the donor of the sugar xylose for the synthesis of cryptococcal capsule, proteins, and lipids. We have identified two UDP-xylose transporters, the first discovered in a fungal organism, and characterized their biochemical properties and role in biology and infection. We found that although this pair of proteins shares biochemical functions, including additional transport of UDP-galactofuranose, the two transporters differ in terms of where they are in the cell, when they are expressed, and their transport properties. Cryptococcal cells lacking both proteins do not cause disease in mice, demonstrating the importance of this synthetic pathway. These findings elucidate fundamental biology as well as fungal pathogenesis.

## Introduction

Glycans are critical for the normal development, growth, and viability of organisms across all kingdoms of life. The extensive glycoconjugate repertoire of *Cryptococcus neoformans*, a ubiquitous environmental fungus, enables this pathogen to cause serious respiratory disease in the setting of immune compromise. This pulmonary infection often progresses to a lethal meningoencephalitis, even with treatment, leading to several hundred thousand deaths each year [1–3].

The major virulence factor of *C*. *neoformans*, a polysaccharide capsule, acts as a physical barrier against host defenses when associated with the cell wall and as an immune modulator when shed into the extracellular space [4,5]. This material consists primarily of two complex polysaccharides, glucuronoxylomannan (GXM) and glucuronoxylomannanogalactan (GXMGal) [4]. The more abundant capsule component, GXM, is a linear mannose (Man) polymer with single glucuronic acid (GlcA) and xylose (Xyl) side chains [6]. The second polysaccharide, GXMGal, consists of a galactose backbone modified with single galactofuranose (Gal*f*) residues and galactomannan side chains bearing a variable number of GlcA and Xyl residues [7–9].

Beyond the capsule, *C*. *neoformans* glycoconjugates include proteins with *N*- and *O*-linked glycans that resemble the corresponding mannose structures of the model yeast *Saccharomyces cerevisiae*, although they are further modified with Xyl or Xyl-phosphate residues [10–13]. Cryptococcal glycosphingolipids range from simple mannose modification of lipids to more complex structures that also incorporate galactose (Gal) and Xyl [14], and the cryptococcal cell wall consists of glucans, chitin, chitosan, and mannoproteins, many of which bear GPI anchors [15]. These glycans play integral structural and regulatory roles to facilitate fungal survival and pathogenesis [16].

Consistent with the abundant glycosylation of *C*. *neoformans*, a significant portion of its genetic machinery and metabolic energy is dedicated to glycan synthesis. These synthetic reactions typically occur in the secretory pathway, although they rely on nucleotide sugar donors that are synthesized in the cytosol [17]. The charged donors enter the luminal space via nucleotide sugar transporters (NSTs), which exchange activated sugars for the corresponding nucleoside monophosphates [18,19]. NSTs thus mediate a limiting step in glycan biosynthesis, and are consequently required for cryptococcal viability and pathogenicity [20–22].

Our focus is on defining glycan synthesis in *C*. *neoformans*, motivated by its unique biology and critical role in a deadly disease. Cryptococcal NSTs comprise a key subset of this machinery, which has stimulated us to identify these proteins and their functions. This effort is complicated by the observations that NST homology is not always a reliable predictor of substrate specificity and that NSTs may be functionally redundant. Individual NSTs also range from highly specific single-substrate transporters to more promiscuous, multi-substrate proteins [23–28]. NST substrate specificity may also be modulated by localization to a particular cellular compartment and/or association with other glycan biosynthetic enzymes [21,29].

In prior work, protein structure predictions and homology facilitated identification of the cryptococcal NSTs responsible for GDP-Man [21,30] and UDP-Gal [20,22] transport. We have now used product analysis and mass spectrometry based assays to discover Uxt1 and Uxt2, which both transport UDP-Xyl and UDP-Gal*f* although they exhibit distinct subcellular localization, expression patterns, and kinetic parameters. Cells without these two proteins lack Xyl in all analyzed glycoconjugates and exhibit growth defects and metabolic abnormalities that are present to a lesser extent in single mutant strains. We further made the unexpected finding that transporter function is required for virulence in a mouse model of disease, but not for persistence in that context.

## Results

In examining the cryptococcal genome for genes encoding putative NSTs, we discovered a pair of sequences (CNAG_02036 and CNAG_03695) that encoded closely related proteins (57% identity at the amino acid level; S1A Fig). We were interested in these sequences because the level of identity could indicate functional redundancy to ensure the transport of a key glycan precursor, or, in the absence of shared activity, could shed light on mechanisms of substrate specificity.

**Fig 1:**
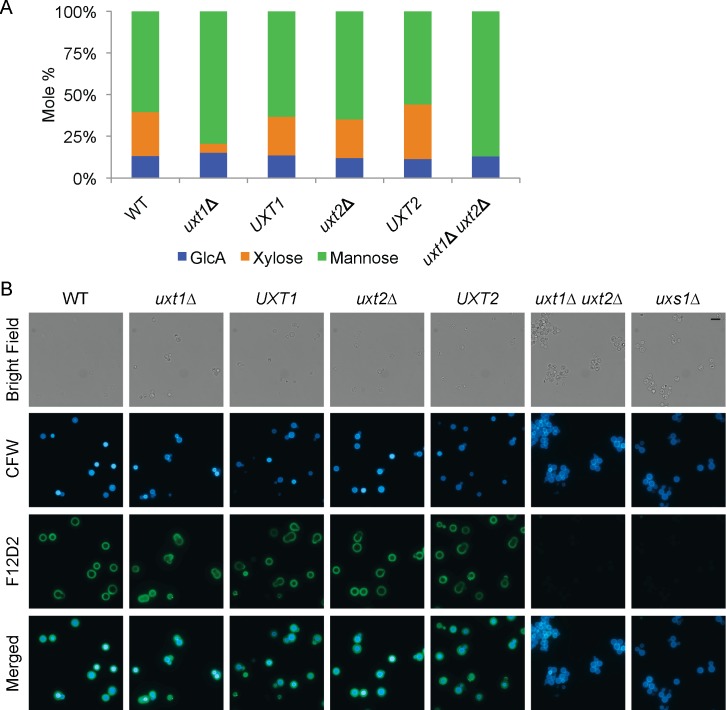
Capsule characteristics of *uxt* mutants. (A) Glycan composition of GXM. (B) Cell wall and capsule staining with Calcofluor white (CFW; blue) and anti-GXM mAb F12D2 (green), respectively. Bright field, single channel, and merged images are shown; scale bar = 10 μm.

To assess the biological role of the novel protein pair we had identified, we generated single and double deletion strains. We first tested whether these mutations affected the major cryptococcal virulence factor, its polysaccharide capsule. Composition analysis of capsule GXM showed complete loss of Xyl from the double mutant, while single mutants were less affected (Fig 1A). This suggested that both proteins transported the Xyl precursor UDP-Xyl, so we designated them as UDP-Xyl transporters 1 and 2 (Uxt1 and Uxt2). *uxt1*Δ GXM had only 20% of the Xyl found in WT material, while *uxt2*Δ exhibited no defect in composition. Consistent with these results, linkage analysis of GXM mannose residues showed a dramatic shift to less substitution of the mannose backbone in the double mutant, with a slightly lesser shift in *uxt1*Δ (S1 Table).

To further examine the mutant capsules, we used a Xyl-dependent monoclonal antibody to GXM [31]. This antibody, F12D2, labeled both single mutant strains, but not *uxt1*Δ *uxt2*Δ (Fig 1B). In this respect the double mutant resembled *uxs1*Δ, a strain that does not synthesize UDP-Xyl [32]. Both *uxt1*Δ *uxt2*Δ and *uxs1*Δ still bind Xyl-independent anti-GXM monoclonal antibodies (S2 Fig, S2 Table).

**Fig 2:**
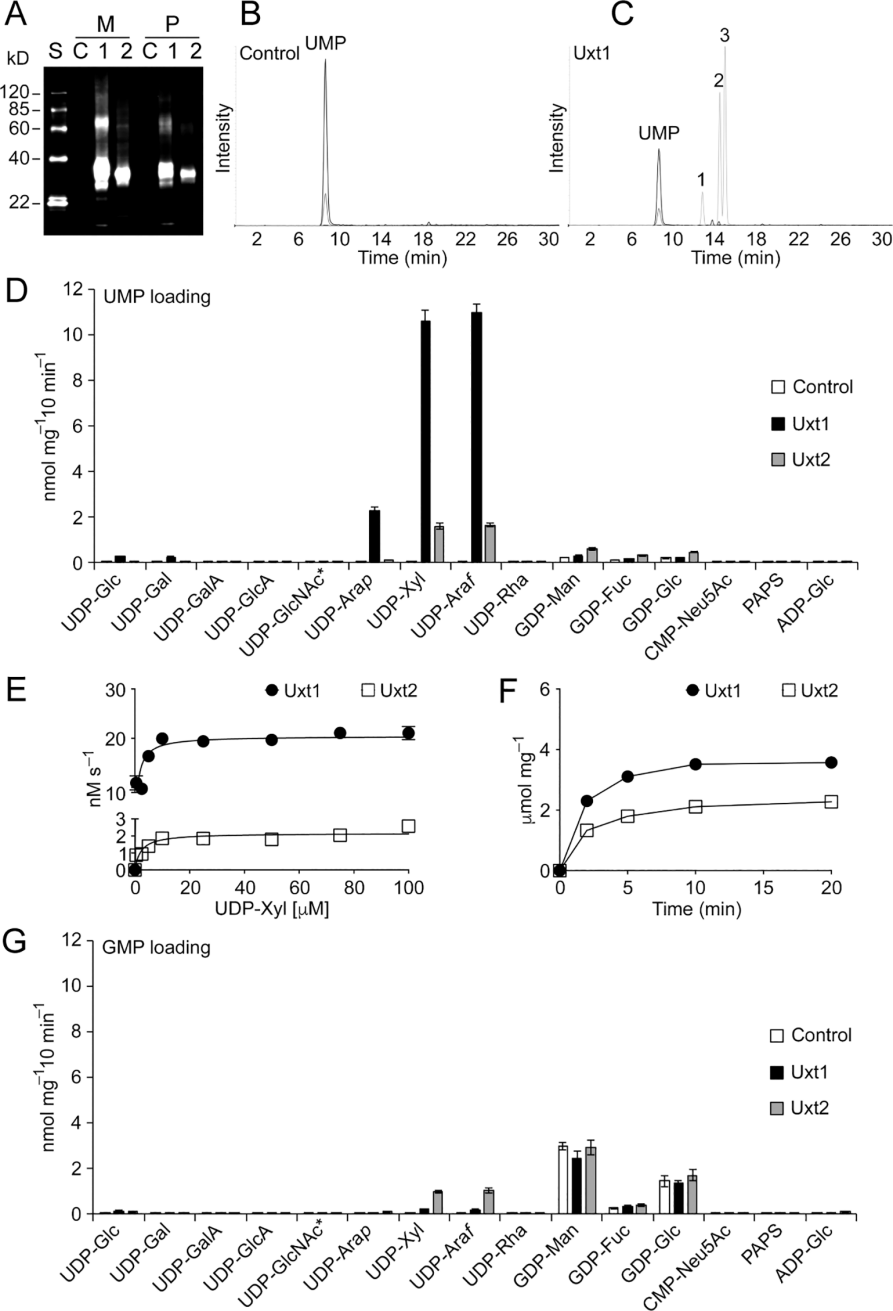
Uxt1 and Uxt2 *in vitro* transport activities. (A) Immunoblot analysis of microscomes (M) and proteoliposomes (P) prepared from *S*. *cerevisiae* expressing vector alone (Control) or V5-tagged Uxt1 or Uxt2 (2.5 μg protein per lane; S, standards; C, control; 1, Uxt1; 2, Uxt2). (B and C) Representative LC-MS/MS spectra of proteoliposomes prepared from (B) control or (C) Uxt1-expressing *S*. *cerevisiae* cells, preloaded with 30 mM UMP, and incubated with a mixture of 16 nucleotide / nucleotide sugar substrates (50 μM each, 10 min, 37°C); Peak 1, UDP-Ara*p*; Peak 2, UDP-Xyl; Peak 3, UDP-Ara*f*. (D) Nucleotide sugar uptake into proteoliposomes preloaded with 30 mM UMP. Values were normalized to the total protein content of the proteoliposome preparations. Data represent the mean ± SD of n = 4 assays. *, mixture of UDP-GalNAc and UDP-GlcNAc. (E and F) Proteoliposomes preloaded with 10 mM UMP were incubated for 2 min with UDP-Xyl (E) at variable concentrations (0–100 μM) or (F) for the indicated times with 50 μM UDP-Xyl. Values were normalized to the actual NST content in proteoliposome preparations (S4 Table). Data are the mean ± SEM of n = 4 assays. (G) Nucleotide sugar uptake into proteoliposomes preloaded with 30 mM GMP analyzed as in (D).

We next used an unbiased approach to directly measure UDP-Xyl transport activity and assay for additional transport substrates. To do this, we prepared proteoliposomes from *S*. *cerevisiae* heterologously expressing Uxt1 and Uxt2 (Fig 2A). When these were preloaded with UMP we observed import of UDP-Xyl (Fig 2B–2D), consistent with our composition studies and antibody binding results. Transport of UDP-Xyl by both proteins was saturable with substrate concentration (Fig 2E) and time (Fig 2F). Uxt1 had an apparent *K*_M_ of 1.0 ± 0.2 μM and V_max_ of 20.4 ± 0.6 nM s^-1^ (mean ± SEM of n = 4) with a turnover rate of 0.9 s^-1^, while Uxt2 exhibited lower affinity and catalytic efficiency with an apparent *K*_M_ of 2.2 ± 0.5 μM, V_max_ of 2.2 ± 0.1 nM s^-1^, and a turnover rate of 0.4 s^-1^. These *K*_M_ values were consistent with the estimated μM physiological concentration of UDP-Xyl (S3 Table).

We further observed transport of UDP-Gal*f*, the donor of a known capsule component, although assessment of its transport kinetics was hindered by its instability, which necessitates simultaneous synthesis and assay. We also observed transport of UDP-Ara*p* and UDP-Ara*f* (Fig 2C and 2D; S3 Fig), although arabinose has never been reported in *C*. *neoformans*. Neither of these donor molecules was detected in our nucleotide sugar analyses (S3 Table).

Surprisingly, Uxt2 was almost as efficient in using GMP as UMP as an antiport substrate for UDP-Xyl and UDP-Gal*f*. In contrast, we observed minimal transport activity over control when Uxt1-bearing proteoliposomes were preloaded with GMP (Fig 2G, S3E Fig). Although Uxt1 and Uxt2 have similar activity, they are clearly not functionally identical at the enzymatic level.

We wondered how Uxt1 and Uxt2, the first reported fungal UDP-Xyl/UDP-Gal*f* transporters, compared to other NSTs with similar substrate specificities. Phylogenetic analysis with known transporters of UDP-Xyl and UDP-Ara*f* placed Uxt1 and Uxt2 closest to the *A*. *thaliana* UDP-Ara*f* transporters (UAfT1-4) even though, as mentioned above, arabinose has never been detected in *C*. *neoformans* (S1B Fig). Interestingly, Uxt1 and Uxt2 were more divergent from known UDP-Xyl transporters, such as those from human and *A*. *thaliana* (S1B Fig), which may be of therapeutic relevance.

**Fig 3:**
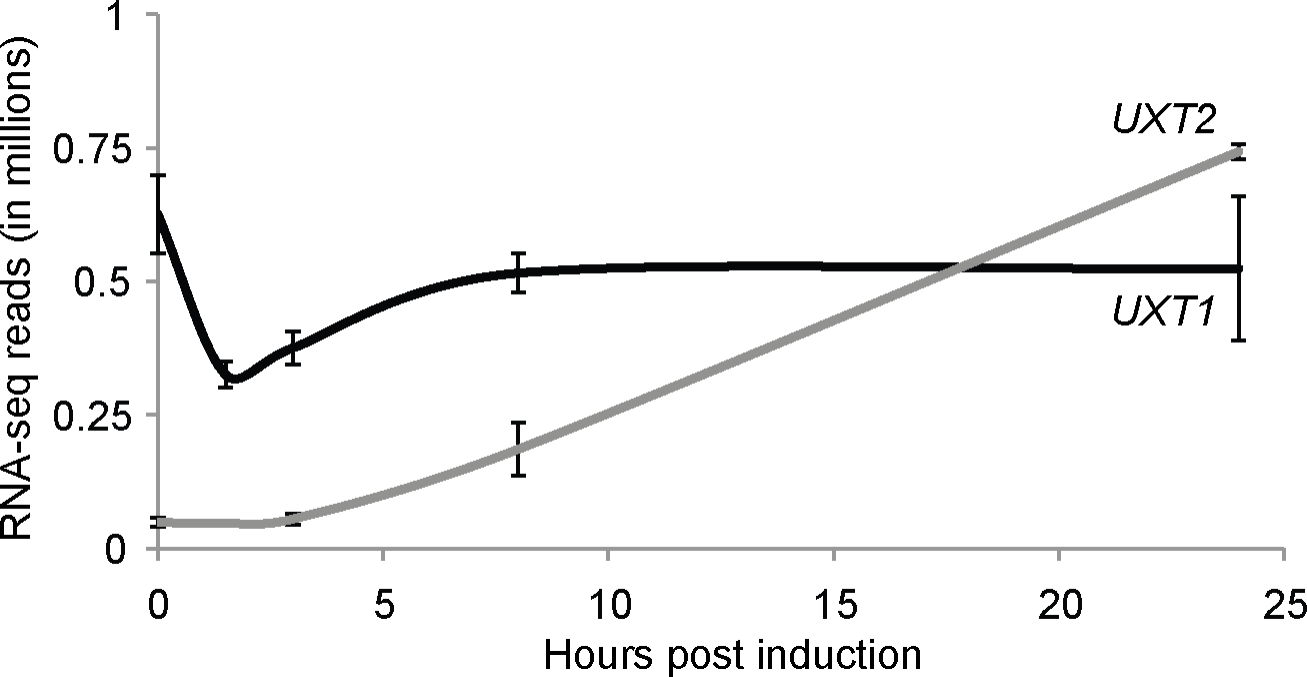
Transcription of *UXT2* but not *UXT1* increases during capsule induction. Reads from RNA-Seq data (mean ± SD) during capsule induction (see Materials and Methods) were compiled from three independent experiments, each with RNA prepared from three biological replicates as in [56].

Our biochemical and phylogenetic studies did not explain why *C*. *neoformans* has two transporters for UDP-Xyl and UDP-Gal*f*, and raised the question of whether they have distinct roles *in vivo*. To define the physiological roles of Uxt1 and Uxt2, we first examined the expression of *UXT1* and *UXT2* under nutrient rich and deficient (capsule-inducing) conditions; the latter was tested because of the central role capsule plays in virulence and the differences we had noted in capsule composition. We found that *UXT1* expression was not affected by capsule induction, while *UXT2* had a lower basal level of expression in rich media (0 h) that was upregulated 15-fold upon capsule induction (Fig 3).

**Fig 4:**
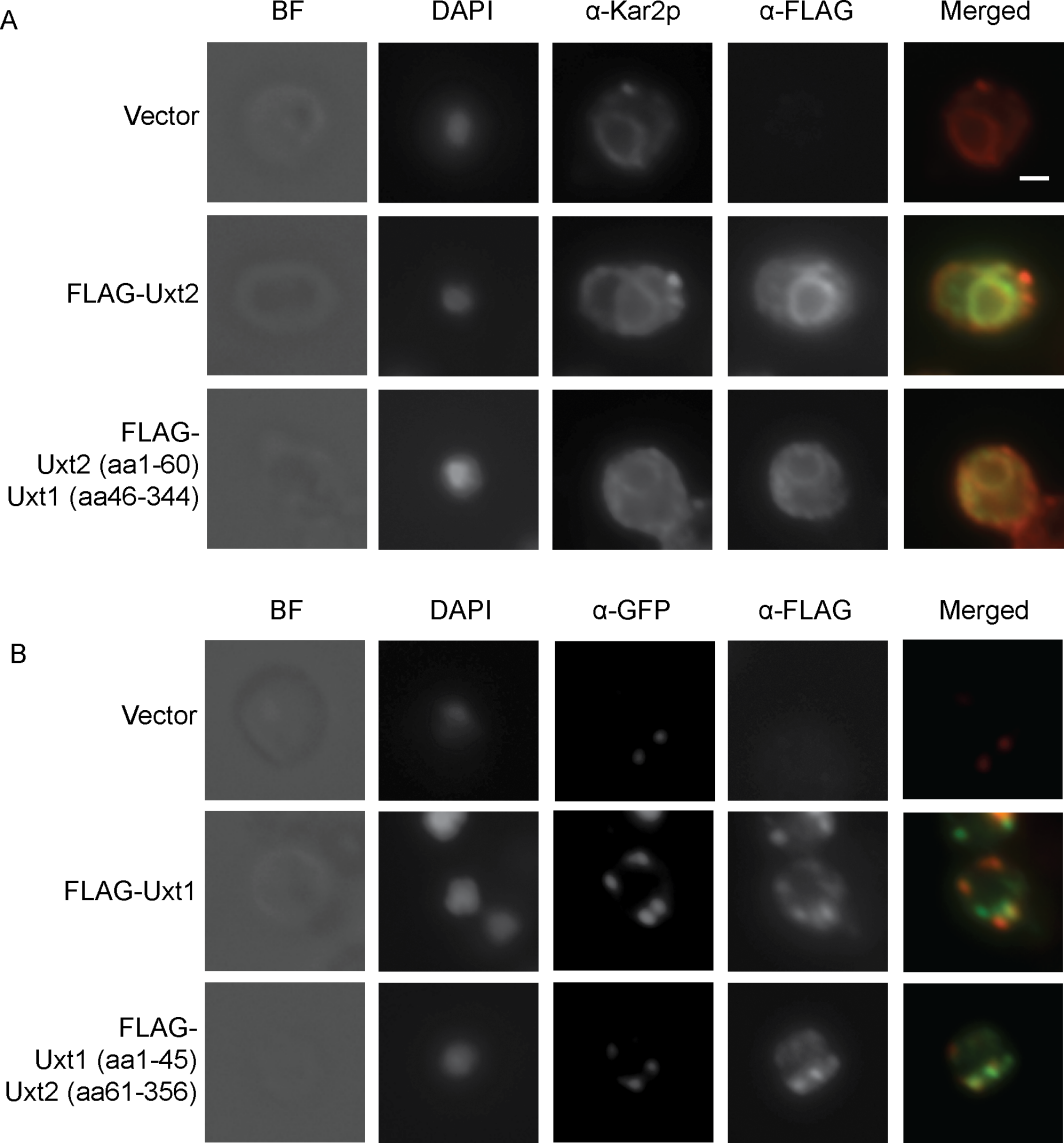
Subcellular localization of Uxt1 and Uxt2. Sec7-3xGFP *S*. *cerevisiae* cells transformed with vector alone (Vector) or vector expressing FLAG-tagged Uxt1, Uxt2, or chimeras of Uxt1 and Uxt2 were stained with DAPI and probed with the indicated antibodies. Bright field, single channel, and merged images are shown (scale bars, 1 μm). Blue, DAPI; red, α-Kar2p/BiP to mark the ER (A) or α-GFP to localize the Golgi marker Sec7 (B); green, α-FLAG. Images are representative of three independent studies.

When we expressed FLAG-tagged Uxt1 and Uxt2 in *S*. *cerevisiae* to assess their subcellular localization, we found that Uxt2 localized to the ER (Fig 4A). In contrast, Uxt1 exhibited a Golgi distribution (Fig 4B), consistent with its predicted N-terminal ER export signal (two di-acidic motifs). Swapping the N-terminal cytosolic domains of the two proteins caused each to shift to the other secretory compartment (Fig 4, bottom row of each panel).

**Fig 5:**
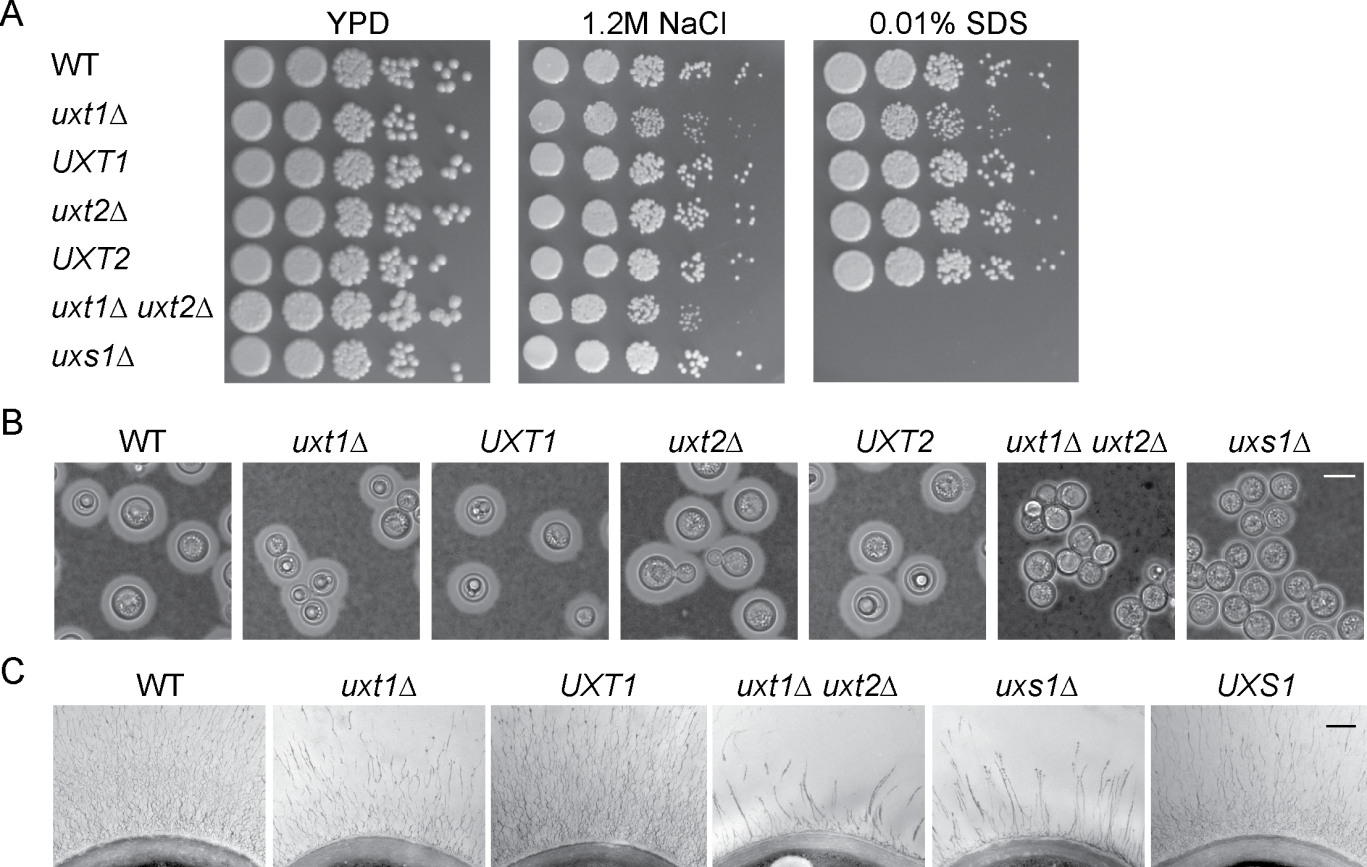
*uxt1*Δ and *uxt1*Δ *uxt2*Δ mutants exhibit growth and capsule defects. (A) 5-fold serial dilutions of the indicated strains, grown on the indicated media at 37°C and photographed after three days. *uxs1*Δ is included as a control. (B and C) The indicated strains were placed in capsule-inducing conditions (see Materials and Methods) for 24 h, and then visualized by light microscopy after negative staining with India Ink (B, scale bar = 5 μm) or by electron microscopy (C, scale bar = 0.5 μm). Additional EM images are provided in S5D Fig.

We wondered if the observed differences in protein expression and localization had phenotypic consequences beyond alterations in GXM. All of the mutants grew normally at 37°C, except for a modest increase in the doubling time of *uxt1*Δ *uxt2*Δ, which was further exacerbated by nutrient limitation (S4 Fig). We saw no changes in growth when these strains were challenged with stressors that target the cell wall, consistent with their wild-type patterns of cell wall staining (S2 Table). At this temperature, however, *uxt1*Δ *uxt2*Δ growth was abolished by SDS (that of *uxt1*Δ was slightly inhibited), and the growth of both of these strains was slightly inhibited by high salt (Fig 5A).

Both single *uxt* mutants showed normal capsule thickness (Fig 5B; S5A Fig), cell diameter (Fig 5B, S5B Fig), and GXM shedding (S5C Fig). The *uxt1*Δ cells, however, aggregated more than wild type (Fig 5B), and differed from wild-type cells in capsule organization, despite the similarity in overall capsule radius: individual fibers seemed thicker and appeared to form a sparser network over the cell surface (Fig 5C, S5D Fig). The capsule changes were more striking in *uxt1*Δ *uxt2*Δ cells; these showed significantly thinner capsules (S5A Fig) and reduced GXM shedding (S5C Fig). Their capsule fibers also appeared shorter and coarser than those of *uxt1*Δ, resembling those of *uxs1*Δ cells, which do not synthesize UDP-Xyl (Fig 5C, S5D Fig).

The observed differences in capsule did not explain the increased sensitivity of *uxt1*Δ and *uxt1*Δ *uxt2*Δ to stress, because even acapsular cells grow normally under these conditions [33,34]. We hypothesized that this sensitivity instead results from reduced Xyl in other glycoconjugates, such as protein-linked glycans. In support of this idea, the Xyl content of glycoproteins isolated from *uxt1*Δ and *uxt2*Δ was 15% and 90% of their respective complements. We detected no Xyl in samples purified from *uxt1*Δ *uxt2*Δ or the control *uxs1*Δ.

**Fig 6:**
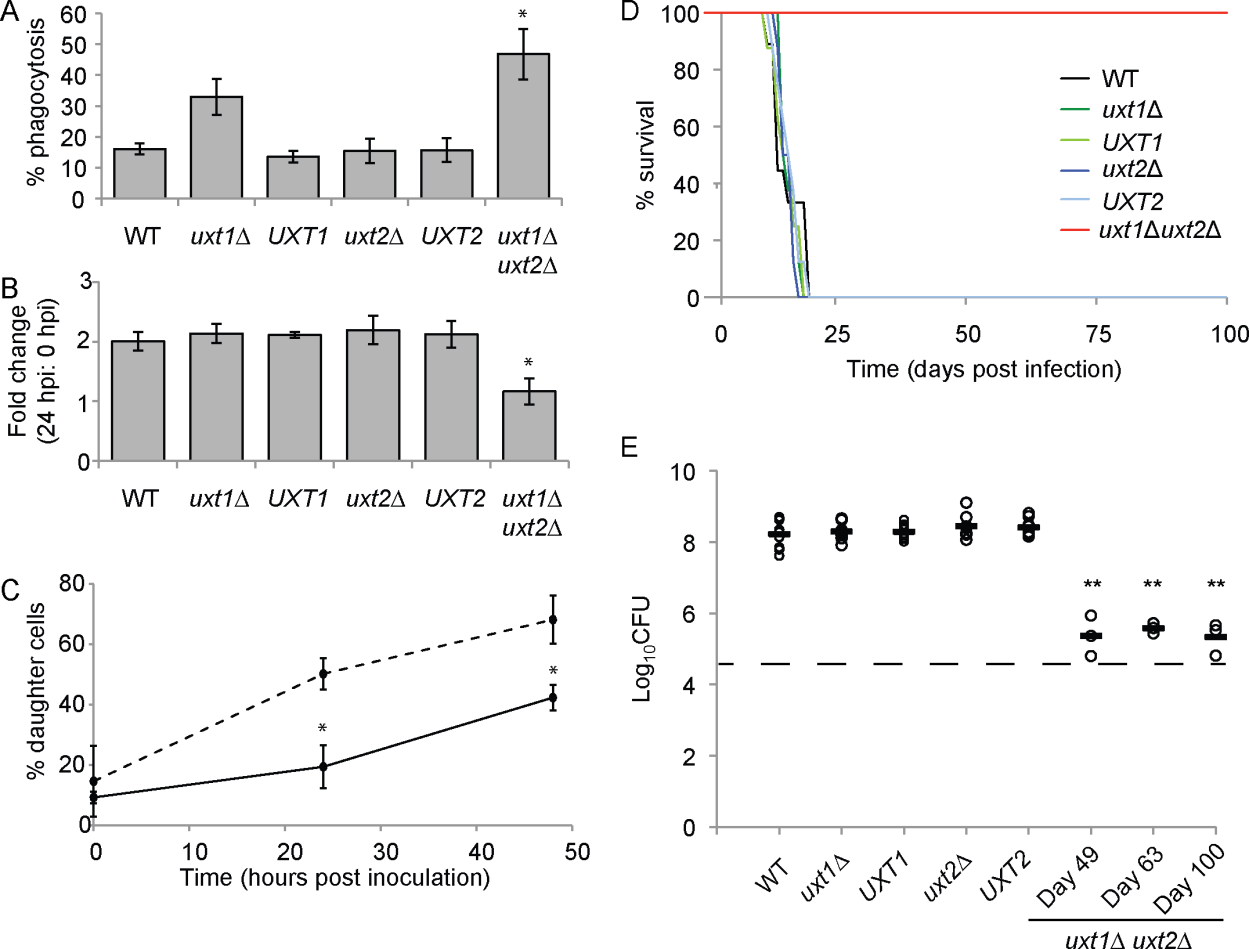
UDP-Xyl transport is required for host interactions and virulence. (A) Percent phagocytosis (engulfed fungi/initial inoculum) of opsonized fungi. (B) Fold-change in colony-forming units (CFU) 24 h:0 h after internalization. (C) Proportion of daughter cells in the population of WT (dashed line) and *uxt1*Δ *uxt2*Δ (black line) cells incubated with BMMs for 0, 24, and 48 h. Data are the mean ± SEM of three independent experiments. *, *p* < 0.05 by (A, B) one-way ANOVA with Tukey’s post hoc test or (C) Student t-test. (D) Survival of A/JCr mice after intranasal inoculation with 5 × 10^4^ cells of the indicated strains (n = 8–9). (E) Lung CFU of infected mice at the time of death (for WT, *uxt1*Δ, *uxt2*Δ, and complemented mutants; n = 8) or at the indicated time points (for *uxt1*Δ *uxt2*Δ; n = 3). Open circles, individual mice; black bar, mean; dashed line, initial inoculum. **, *p* < 0.01 by one-way ANOVA with Tukey’s post hoc test.

We wondered whether the stress sensitivity and altered glycoconjugate xylosylation of the *uxt* mutants would translate into aberrant interactions with host cells. Since host macrophages are critical for determining the outcome of cryptococcal infection [35], we investigated the ability of our mutants to interact with bone marrow macrophages (BMM) *in vitro*. We found that the level of internalization by BMMs was inversely related to the degree of xylosylation: *uxt1*Δ *uxt2*Δ was taken up more readily than WT cells while *uxt1*Δ exhibited an intermediate phenotype (Fig 6A). Notably, while WT and the single deletion strains replicated ~2-fold over 24 h after internalization by BMM, the level of *uxt1*Δ *uxt2*Δ did not change (Fig 6B). This reflected both decreased replication and increased clearance, which negated the small growth that occurred (Fig 6C).

The altered host interactions we observed *in vitro* suggested a potential defect in pathogenicity of the *uxt* strains. Studies using an inhalational model to mimic the natural route of infection showed that *uxt2*Δ and, more surprisingly, *uxt1*Δ, caused disease with normal kinetics (Fig 6D) and organ burdens (Fig 6E, S7 Fig). In contrast, *uxt1*Δ *uxt2*Δ was attenuated for virulence in both A/JCr and C57BL/6 mice (Fig 6D, S6 Fig). More detailed studies using A/JCr mice showed that the double mutant was unexpectedly detectable in the lungs out to 100 days post infection (dpi), when the experiment was terminated (Fig 6E). Despite the persistent pulmonary burden, *uxt1*Δ *uxt2*Δ failed to disseminate from the lungs; it was never detected in the spleen and was only transiently detected in the brain (S7 Fig).

## Discussion

*C*. *neoformans* encodes an unusual pair of highly homologous UDP-Xyl/UDP-Gal*f* transporters, which together are critical for virulence. Uxt1 and Uxt2 are unique for their high affinity for UDP-Xyl (Fig 2), with *K*_M_ values almost two orders of magnitude lower than those of the *Arabidopsis* UDP-Xyl transporters [36]. Despite transporting the same nucleotide sugars, the two proteins are not completely functionally redundant, likely due to differences in expression, enzyme kinetics, and localization.

Beyond nucleotide sugars known to occur in *C*. *neoformans*, *in vitro* Uxt1 and Uxt2 also transport UDP-Ara*p* and UDP-Ara*f* (Fig 2), potentially enabled by the similar structures of Xyl and Ara (D-Xyl and L-Ara are epimers). While the NSTs most closely related to Uxt1 and Uxt2 (*At* UAfT1-4) are highly specific for UDP-Ara*f* [37], the substrate range of the cryptococcal proteins most closely resembles that of plant UDP-Xyl transporters (UXT1-3;[36]) despite their sequence divergence (S1B Fig). These observations highlight the importance of using rigorous biochemical analysis to test functional assumptions based on homology.

Since UDP-Ara is not found in cryptococcal cells and abrogating UDP-Gal*f* synthesis does not alter cryptococcal growth or virulence [8], the phenotypes associated with loss of Uxt1 and Uxt2 likely result from disruption of UDP-Xyl transport into the secretory compartment. Notably, capsule material was still produced (Fig 5) even when no Xyl was detected in GXM because both transporters were absent (Fig 1A). This suggests that Xyl incorporation is not required for GXM backbone synthesis or elongation, or for incorporation of GlcA. However, lack of the Xyl donor did reduce the amount of shed capsule material by over 75% (S5C Fig). Since Xyl constitutes only 20–30% of the capsule mass, loss of this moiety alone does not explain this reduction. Instead, it may be a direct effect of the reduced Xyl incorporation, if these side chains are needed for capsule recognition by synthetic or trafficking machinery, or an indirect effect, for example if synthetic enzymes must be xylosylated to function efficiently. Lack of UDP-Xyl transport also yielded thinner capsules (Fig 5B, S5A Fig) with abnormal fiber morphology (Fig 5C, S5D Fig); this presumably results from the lack of Xyl substitution, which may be required for proper conformation or organization of capsule polysaccharides.

Why does *C*. *neoformans* express two UDP-Xyl transporters? Judging by the severity of mutant phenotypes (Fig 5) and the gene expression levels (Fig 3), Uxt1 is the major transporter of the pair, but loss of both is required to eliminate Xyl incorporation (Fig 1). These data exclude the possibility of a third UDP-Xyl transporter of any significance, while highlighting the unequal contribution of these two proteins. One factor in this inequity is likely the higher affinity and catalytic efficiency for UDP-Xyl transport of Uxt1 compared to Uxt2 (Fig 2). Another is probably their distinct regulatory patterns, with *UXT1* expressed constitutively, while *UXT2* expression levels is upregulated in response to greater glycan biosynthetic demands (Fig 3). Curiously, expression of the two genes was not optimally regulated to enable compensation in the single mutants: expression of *UXT1* did not change in response to the loss of *UXT2* even in capsule-inducing conditions, and the normal *UXT2* induction was muted in the absence of *UXT1* (S8 Fig). Future studies will address this regulatory relationship.

The distinct roles of Uxt1 and Uxt2 also potentially reflect their association with other glycan synthetic proteins, such as glycosyltransferases. We found no evidence of association with specific xylosyltransferase(s), as for example preferential loss of β-1,2 or β-1,4 linked Xyl in the GXM of either mutant (S1 Table). However, the full cryptococcal glycan repertoire is not known; future studies may enable us to identify specific protein or lipid modifications enabled by each enzyme. Another factor in the dominant role of Uxt1 is likely its localization to the Golgi (Fig 4B), the probable site of capsule and protein xylosylation [10,11,14], in contrast to the ER localization of Uxt2 (Fig 4A). The latter is intriguing, as this compartment is upstream of most glycan synthesis. It is possible that Uxt2 has transport-independent functions, or that it supplies novel synthetic processes that have yet to be described. These will be exciting areas for future investigation.

The increased sensitivity to stress (Fig 5A) and greater uptake by host phagocytes (Fig 6A) of *uxt1*Δ were insufficient to alter its behavior in animal infection (Fig 6D). We expected the highly impaired double mutant *uxt1*Δ *uxt2*Δ, which cannot transport UDP-Xyl into the secretory pathway, to behave like strains that cannot synthesize UDP-Xyl (*uxs1*Δ), which are avirulent and completely cleared by 7 days post-infection [38]. Surprisingly, this mutant persisted in the lungs (Fig 6E), suggesting either a cytosolic role for UDP-Xyl or a UDP-Xyl-independent role for Uxs1; these possibilities remain to be investigated.

The double mutant population increased very slowly in both A/JCr and C57BL/6 mice, likely due to its slower growth rate under stress (Fig 6C, S4 Fig) and reduced ability to resist host defenses (Fig 6A and 6B). Xyl modifications have been identified as immunodominant epitopes in antibody responses to allergens and pathogens [39,40], and the absence of Xyl modifications in *uxt1*Δ *uxt2*Δ did increase immune detection and clearance of the pathogen *in vitro* (Fig 6A and 6B). The mutant also remained confined to the lungs of A/JCr mice (S7 Fig) and was slow to cause lethal meningoencephalitis in C57BL/6 mice (S6 Fig). This may reflect an inability to disseminate or to efficiently establish infection at distal sites, or may be the result of active restriction by the immune system. Notably, phagocytes have a multifaceted role in cryptococcal infection, potentially aiding and/or inhibiting fungal survival and dissemination depending on the circumstance [35]. Elucidating the complex interplay between Uxt mutants and the infected host will be the focus of future work. Further studies may also uncover facets of this infection that could be exploited for therapeutic intervention and potentially inform vaccine design.

*C*. *neoformans* is unusual among yeast for its extensive utilization of Xyl, in capsule polysaccharides, *N*- and *O*-linked glycans (including a unique Xyl-phosphate modification), and glycoplipids. By elucidating UDP-Xyl transport, we have expanded our understanding of this aspect of cryptococcal glycan biosynthesis, including the sequence and localization of capsule synthetic events, and of NSTs as a protein family. We have identified the first fungal UDP-Xyl/UDP-Gal*f* transporters and also set the stage for studies of an unusual mutant that may help elucidate mechanisms of cryptococcal pathogenesis and host response.

## Materials and methods

### Sequence and phylogenetic analysis

Uxt1 and Uxt2 were identified by BLASTP searches of known NSTs against *C*. *neoformans* predicted proteins (Broad Institute; *Cryptococcus neoformans var*. *grubii* H99 database); the closest related sequence was that of the *Aspergillus fumigatus* UDP-Gal*f* transporter (ACR56866.1). The online Phylogeny.fr program (http://www.phylogeny.fr/.version2_cgi/index.cgi) with default settings [41,42] was used for multiple sequence alignment (MUSCLE; [43]), phylogenetic analysis (PhyML; [44]), and tree rendering (TreeDyn; [45]) of Uxt1 and Uxt2 and other NSTs. These included transporters of UDP-Gal*f* (*Aspergillus fumigatus*, *Af*), UDP-Xyl (*Homo sapiens*, *Hs*, UXT NP_116215.1; *Arabidopsis thaliana*, *At*, UXT1 NP_850120.3 (At2g28315), *At* UXT2 NP_180604.4 (At2g30460), and *At* UXT3 NP_172172.2 (At1g06890)), and UDP-arabinofuranose (*At* UAfT1 NP_568469.1, At5g25400; *At* UAfT2 NP_196684.1, At5g11230; *At* UAfT3 NP_194965.1, At4g32390; *At* UAfT4 NP_180122.1, At2g25520), as well as other cryptococcal (*Cn*) NSTs.

Sequence alignment between Uxt1 and Uxt2 was analyzed using T-coffee (http://tcoffee.crg.cat/apps/tcoffee/do:regular) and formatted using Boxshade (http://www.ch.embnet.org/software/BOX_form.html). The protein sequences were analyzed for predicted localization signals using LocSigDB (http://genome.unmc.edu/.LocSigDB/; [46]).

### Cell growth

*C*. *neoformans* strains were grown in YPD medium (1% w/v BactoYeast Extract, 2% w/v BactoPeptone, 2% w/v dextrose) at 30°C with shaking (230 rpm) unless otherwise noted. For phenotypic analysis, cells were grown overnight (O/N), washed in sterile phosphate buffered saline (PBS), and diluted to 10^6^ cells/mL in PBS. 4 μL aliquots of serial 5-fold dilutions were plated and grown at 30 or 37°C as indicated. The stress conditions tested included YPD containing 0.01% SDS, 1.2 M NaCl, 1.2 M KCl, Tris pH 8.8, 1.5 M Sorbitol, 0.05% Congo Red (CR), or 2% Calcofluor White (CFW). To test oxidative and nitrosative stress sensitivity, dilutions were spotted onto solid YNB medium (0.67% w/v yeast nitrogen base without amino acids, 2% w/v glucose, 2% w/v agar, 25 mM sodium succinate, pH 4.0) supplemented with 0.5 mM hydrogen peroxide (H_2_O_2_) or 0.5 mM sodium nitrite (NaNO_2_). To assess cell-associated melanin production, 5 μL of a 10^6^ cells/mL solution was plated on agar plates containing 8 mg/mL KH_2_PO_4_, 2 mg/mL glucose, 2 mg/mL L-glycine, 1 μg/mL D-biotin, 1μg/mL thiamine, 0.92 mg/mL MgSO_4_ 7H_2_O, and 0.4 mg/mL L-3,4-dihydrohyphenylalanine (L-DOPA; Sigma-Aldrich). To assay growth, cells were cultured O/N; washed in sterile PBS; resuspended at 10^5^ cells/mL in 30 mL of YPD, YNB, DMEM, or RPMI; and incubated at 37°C for 120 h, with triplicate samples counted by hemocytometer at various times.

### *C*. *neoformans* strains

We replaced *UXT1* in KN99α (WT) with a nourseothricin (NAT) resistance marker using a split marker strategy [47]. Transformants of interest were identified by resistance to NAT and validated by PCR verification of gene replacement. We used a similar strategy to complement the *uxt1* deletion strain at the endogenous locus by replacing the deletion cassette with *UXT1* in tandem with a G418 resistance marker. Transformants resistant to G418 and sensitive to NAT were verified by PCR and assessed for reversal of mutant phenotypes (see Results). We generated *uxt2*Δ and *UXT2* with an identical approach, using G418 and NAT markers in the deletion and complement constructs, respectively. To obtain an *uxt1*Δ *uxt2*Δ double mutant, we crossed the single mutants on V8 agar plates [48]. Double mutants were selected for by resistance to both drugs and verified by PCR amplification.

### Capsule induction and visualization

O/N cultures of *C*. *neoformans* were collected by centrifugation, washed twice with sterile PBS, diluted to 10^6^ cells/mL in DMEM and incubated at 37°C in 5% CO_2_ for 24 h in T-75 tissue culture flasks or 24-well plates. The cells were then washed and resuspended in PBS, mixed with 1.5 parts India Ink, and viewed by light microscopy with a ZEISS Axioskop2 MOT Plus microscope (Carl Zeiss Microscopy, LLC).

For antibody detection of cell wall-associated GXM, strains were induced as above for 24 h, fixed for 1 h in 3.7% formaldehyde, washed in PBS, and then incubated for 1 h at room temperature (RT) with 1 mg/mL of anti-GXM monoclonal antibody (mAb) F12D2 or 302 (from Dr. Thomas R. Kozel, University of Nevada School of Medicine) conjugated to AlexaFlour 488. Stained cells were washed twice with PBS, resuspended in PBS, and examined on a ZEISS Axioskop 2 MOT Plus microscope.

### GXM ELISA

GXM content of supernatant fractions from cell cultures was quantified by ELISA according to previous methods [49], using anti-GXM mAb 339 (from Dr. Thomas R. Kozel, University of Nevada School of Medicine).

### Glycan isolation and analysis

GXM was isolated from strains of interest by selective precipitation of culture supernatants with hexadecyltrimethylammonium bromide (CTAB) as detailed in [11]. For isolation of soluble glycoproteins, O/N cultures were diluted into YPD and grown to 10^7^ cells/mL. 2 x 10^7^ cells per strain were collected, washed in Tris-EDTA buffer (100 mM Tris pH 8.5, 0.1 mM EDTA pH 8.0), and resuspended in 40 mL Tris-EDTA buffer with protease inhibitors. Samples were then subjected to 15 cycles of bead beating (3 min) alternating with 3 min on ice, which yielded ~75% cell lysis (as judged by microscopy). All subsequent steps were performed at 4°C. Lysates were collected, pooled with three 10 mL rinses of the beads, and subjected to a clearing spin (1000 x g; 25 min). Supernatant fractions were then transferred to fresh tubes, adjusted to a final concentration of 1% CHAPS, incubated with rocking for 2 h, and subjected to ultracentrifugation (75000 x g; 45 min). The CHAPS extract was then dialyzed (8000 M_r_) against 2 L of 50 mM NH_4_HCO_3_ with three buffer changes over 48 h, lyophilized, and washed with 80% acetone to reduce detergent and polymeric contaminants.

For compositional analysis, per-*O*-trimethylsilyl (TMS) derivatives of monosaccharide methyl glycosides were produced from the GXM samples by acidic methanolysis using methods described in [50,51]. Glycosyl composition was then determined by combined gas chromatography/mass spectrometry (GC/MS) on an Agilent 7890A GC interfaced to a 5975C MSD (mass selective detector, electron impact ionization mode; Agilent Technologies) with a Supelco EC-1 fused silica capillary column (30 m × 0.25 mm ID; Sigma-Aldrich). For linkage analysis, GXM samples were permethylated, depolymerized, reduced, and acetylated as described in [7]. The resultant partially methylated alditol acetates (PMAAs) were then analyzed as above but using a 30 m Supelco SP-2331 bonded phase fused silica capillary column (Sigma-Aldrich).

### Heterologous expression, reconstitution, and transport assays

The *UXT1*, *UXT2*, *GMT1*, and *GMT2* coding regions were amplified from WT cDNA and introduced into the pENTR/SD/D-TOPO vector (Life Technologies) according to the manufacturer’s protocols to generate pENTR-*UXT1*, pENTR-*UXT2*, pENTR-*GMT1*, and pENTR-*GMT2*. Recombination of each entry clone with destination vector pYES-DEST52 (Life Technologies) using LR clonase II (Life Technologies) produced a C-terminal His/V5 epitope fusion that was verified by sequencing before transformation into *S*. *cerevisiae* strain INVSc1 (Thermo Fisher Scientific). Heterologous expression, reconstitution into proteoliposomes, and transport assays were performed as previously described [52]. UDP-Gal*f* was prepared from UDP-galactopyranose (UDP-Gal*p*) according to [53]. Protein expression and incorporation was verified by polyacrylamide gel electrophoreses and immunoblot analysis of 2.5 μg of microsomes or proteoliposomes using anti-V5 antibody (Thermo Fisher Scientific), also as previously described [52]. Kinetic parameters were calculated by non-linear regression using the Prism 6 application (GraphPad Sofware). The assay was validated and its sensitivity confirmed using the well-characterized GDP-Man transporters Gmt1 and Gmt2 (S9A Fig). Both proteins transported GDP-Man and smaller amounts of other GDP-sugars in exchange for GMP and, significantly less efficiently, UMP (S9D and S9E Fig).

### Nucleotide sugar measurement

Nucleotide sugars were extracted from approximately 50 mg of ground cells (wet weight) as previously described [54]. Four biological replicates were processed per strain and condition, and then analyzed in duplicate by LC-MS/MS using porous graphitic carbon as the stationary phase on an 1100 series HPLC system (Agilent Technologies) and a 4000 QTRAP LC/MS/MS system (Sciex) equipped with a TurboIonSpray ion source as in [55]. Results in pmol mg^-1^ wet weight were converted to concentrations using a cell volume of 47.7 μm^3^ (based on the average radius of 10^7^ cells, measured by cellometer (Nexcolom Bioscience LLC; n = 3)) and a mass of 4.35 x 10^−8^ mg/cell (based on weighing a known number of cells; n = 3).

### Protein localization

For expression in *S*. *cerevisiae*, *UXT1* and *UXT2* were amplified from WT cDNA, cloned into the copper-inducible expression vector pYEScupFLAG*K* [26], and transformed using lithium acetate into *S*. *cerevisiae* strain Sec7-3xGFP (from Dr. Benjamin S. Glick, University of Chicago). To generate N-terminal swaps of Uxt1 and Uxt2, we amplified both genes from the start codon to the beginning of the first predicted transmembrane domain (*UXT1* bp 1–135, *UXT2* bp 1–180) and from the first transmembrane domain until the stop codon (*UXT1* bp 136–1032; *UXT2* bp 181–1068), using WT cDNA as a template. We then PCR amplified to fuse the N-terminal region of *UXT1* to the transmembrane region of *UXT2* and vice versa, cloned each construct into pYEScupFLAG*K*, and transformed into *S*. *cerevisiae* Sec7-3xGFP as above. All constructs were verified by sequencing.

For localization, cultures were grown O/N in synthetic complete media without uracil (SC-URA), adjusted to OD 0.5 and 0.5 mM CuSO_4_, and cultured for an additional hour. The cells were then fixed for 30 min in 1% paraformaldehyde, washed and resuspended in 0.1M KPO_4_/1.2 M sorbitol, and incubated for 15 min in the same buffer containing β-mercaptoethanol and zymolase (100 μg/mL). 15 μL of the cells were then spotted onto polylysine-coated slides (Electron Microscopy Sciences), incubated for 10 min, and plunged into methanol for 5 min followed by acetone for 30 sec. The samples were blocked with 5% goat serum in PBS for 30 min, and stained O/N at 4°C with anti-FLAG (Mouse, 1:1000; Invitrogen) and anti-Kar2p/BiP antibody (Rabbit, 1:1000; from Dr. Jeff Brodsky, University of Pittsburgh). Finally, cells were incubated for 2 h with AlexaFluor 594-tagged goat anti-mouse IgG, AlexaFluor 488-tagged goat anti-rabbit IgG (Thermo Fisher Scientific), and DAPI (Thermo Fisher Scientific), and viewed with a ZEISS Axioskop2 MOT Plus microscope.

### Fungal gene expression

Wild-type cells cultured O/N in YPD were placed in DMEM capsule-inducing conditions and sampled at 0, 1.5, 3, 8, and 24 h for RNA isolation and sequencing as in [56]. Additional samples were collected at 0 and 24 h for qPCR analysis. Levels of *UXT1*, *UXT2*, and the reference gene *ACT1* were quantified using the CFX96 Real Time System (BioRad). All sample reactions contained 1 μL cDNA (100 ng), 4 μL of each primer (200 nM), and 10 μL SYBR Select Master Mix (Applied Biosystems). qRT-PCR was performed in triplicate for each sample and non-template controls (for each set of primers) using 15 min activation and denaturation at 95°C followed by 40 cycles of 15 sec at 95°C, 30 sec at 60°C, and 30 sec at 72°C. Baseline and threshold values were determined for all reactions using CFX manager software (BioRad) and exported to Microsoft Excel for additional analysis using the Δ*C*_*q*_ method.

### Electron microscopy

Strains were induced for capsule (as above), collected by centrifugation, fixed for 1 h at RT with 2% glutaraldehyde (Polysciences Inc.) in 100 mM phosphate buffer (pH 7.2), and incubated for 1 h in 1% osmium tetraoxide (Polysciences Inc.). Cells were then dehydrated with ethanol and propylene oxide and embedded in Eponate 12 resin (Tel Pella Inc.). 70 to 90 nm sections were cut with an UCT ultramicrotome (Leica Microsystems Inc.) and stained with uranyl acetate and lead citrate for visualization with a JOEL 1200EX transmission electron microscope (Joel Inc.).

### Macrophage assays

Bone marrow (BMMs) from the femurs and tibiae of C57BL/6 mice (Jackson Laboratory) was incubated for one week at 37°C and 5% CO_2_ in BMM medium (20% FBS, 30% L-cell supernatant, 1% Penicillin-Streptomycin in RPMI), which was refreshed 4 and 6 days after plating. Cells were harvested on day 7 by incubation in ice-cold PBS for 10 min and BMM were purified from the population by positive selection using biotinylated α-F4/80 antibody (eBioscience) and anti-biotin conjugated magnetic beads (Miltenyi Biotec). BMMs were then plated in 24-well plates at 3.5 x 10^5^ cells/mL of R10 media, and incubated O/N at 37°C and 5% CO_2_. On the following day, log-phase fungi were collected by centrifugation, washed, and opsonized with mouse serum (40%) for 30 min at 37°C. The strains were then washed with PBS, resuspended at 3.5 x 10^4^ cells/mL in DMEM, and incubated with macrophages for 1 h. Samples were washed twice with PBS, and lysed using water either immediately or after 24 h incubation in DMEM at 37°C and 5% CO_2_. For CFU quantification, the lysates and initial inocula were plated on YPD agar. Results were analyzed using one-way analysis of variance (ANOVA) with Tukey’s *post-hoc* test. For assays distinguishing parental and daughter cells, fungi were also stained with Oregon Green 488 dye (2 μg/mL; ThermoFisher) in 0.1 M sodium bicarbonate (pH 8.0) for 1 h at room temperature prior to opsonization and then treated as described above. Following lysis, cells were stained with calcofluor white (2 mg/mL PBS) for 30 min before flow analysis with a BD LSRFortessa X-20 using OneComp eBeads (eBioscience) for compensation controls. Data were analyzed using FlowJo (Treestar) and compared using Student’s t-tests.

### Animal studies

Fungal strains were cultured O/N in YPD, washed, and diluted to 10^6^ cells/mL in sterile PBS. 50 μL aliquots of each strain were inoculated intranasally into groups of eight 6- to 8-week-old female A/JCr (National Cancer Institute) or C57BL/6 (Jackson Laboratory) mice. Infected mice were weighed daily and sacrificed if they lost >20% relative to peak weight, or on day 49, 63, or 100 post infection, whichever came first. Lung, brain, and spleen homogenates were harvested and plated for CFU at time of death or indicated time points, and organ burdens were analyzed by ANOVA with Tukey’s *post-hoc* test.

### Ethics statement

All animal studies were approved by the Washington University Institutional Animal Care and Use Committee (Protocol #20140184). All research involving animals was carried out in strict accordance with the “Guide for the Care and Use of Laboratory Animals” published by the National Research Council and endorsed by the Association for the Assessment and Accreditation of Laboratory Animal Care.

## Supporting information

S1 Fig Conservation of cryptococcal nucleotide sugar transporters. (A) Protein sequence alignment of Uxt1 and Uxt2 (CNAG_02036 and CNAG_03695) with conserved residues highlighted (black, identical residues; grey, conserved substitutions). (B) Phylogenetic relationships of *C*. *neoformans* (*Cn*) NSTs (including Uxt1 and Uxt2, in bold), and UDP-Xyl, UDP-Gal*f*, and UDP-Ara*p* transporters from other organisms (*Hs*, *Homo sapiens*; *Af*, *Aspergillus fumigatus*; *At*, *Arabidopsis thaliana*) using MUSCLE, PhyML, and TreeDyn software (see Materials and Methods). Branch lengths are drawn to scale.
(PDF)

S2 Fig *uxt1*Δ *uxt2*Δ is recognized by Xyl-independent capsule antibodies. Cells from the indicated strains were incubated with calcofluor white (CFW; blue) to stain the cell wall and anti-GXM mAb 302 to visualize the capsule (green). Bright field, single channel, and merged images are shown; scale bar = 10 μm. *cap59*Δ is an acapsular strain included as a control.
(PDF)

S3 Fig Uxt1- and Uxt2-mediated UDP-Gal*f* uptake into proteoliposomes. (A) LC-MS/MS analysis of UDP-Gal*f* prepared from UDP-Gal*p* utilizing *E*. *coli* UDP-galactopyranose mutase (GLF). (B-D) Proteoliposomes prepared from *S*. *cerevisiae* expressing vector alone (B), Uxt1 (C), or Uxt2 (D) were preloaded with 30 mM UMP, and analyzed by LC-MS/MS after a 10 min incubation with 700 μM UDP-Gal*p* and 10 μg purified GLF. Based on mass and retention time, the minor peak between UDP-Gal*p* and UDP-Gal*f* is likely UDP-Glc, presumably present in the reaction starting material. (E and F) Quantification of nucleotide sugar uptake into proteoliposomes preloaded with 30 mM UMP (E) or 30 mM GMP (F). Amounts were calculated using a UDP-Gal*p* standard and normalized to the total protein content of the proteoliposome preparations and the mean ± SD of four assays are plotted. All assays were performed at 37°C.
(PDF)

S4 Fig *uxt1*Δ *uxt2*Δ growth is restricted at 37°C. The indicated *C*. *neoformans* strains were grown overnight at 30°C in YPD, diluted to 10^5^ cells/mL in the media indicated, and incubated at 37°C with 5% CO_2_. The results shown are the averages of three measurements. Black, WT; red, *uxt1*Δ; green, *UXT1*; purple, *uxt2*Δ; blue, *UXT2*; grey, *uxt1*Δ *uxt2*Δ (continuous and dashed lines, representing three independently obtained double deletion strains).
(PDF)

S5 Fig Morphological defects of *uxt1*Δ *uxt2*Δ. Induced cells were stained with India Ink, and the radius of the capsule (A) and diameter of the cell body (B) were measured using ImageJ (100 cells counted per strain; mean ± SEM of three biological replicates). (C) GXM shed from equal numbers of each of the indicated strains was quantitated by ELISA (see Materials and Methods). Data is the mean ± SEM of three independent experiments. *, *p* < 0.05, one-way ANOVA with Tukey’s *post-hoc* test. (D) Electron micrographs of the indicated strains induced for capsule as in Fig 5. Two representative images are displayed for each strain. Scale bar = 0.5 μm.
(PDF)

S6 Fig *uxt1*Δ *uxt2*Δ is severely attenuated for virulence in C57BL/6 mice. Survival of C57BL/6 mice after intranasal inoculation with 5 × 10^4^ cells of WT (n = 5) or *uxt1*Δ *uxt2*Δ (n = 19). C57BL/6 mice naturally skew towards a non-protective Th2-type response, which increases their susceptibility to cryptococcal infection compared to A/JCr mice [57].
(PDF)

S7 Fig *uxt1*Δ *uxt2*Δ does not colonize extrapulmonary sites. Brain (A) and spleen (B) CFU of infected A/JCr mice at the time of death (for WT, *uxt1*Δ, *uxt2*Δ, and complemented mutants; n = 8) or at the indicated time points (for *uxt1*Δ *uxt2*Δ; n = 3). Open circles, individual mice; black bar, mean; dashed line, initial inoculum. **, *p* < 0.01 by one-way ANOVA with Tukey’s post hoc test.
(PDF)

S8 Fig *UXT1* and *UXT2* transcription levels. Expression of *UXT1* and *UXT2* measured by qRT-PCR with RNA prepared from the indicated strains after growth in nutrient rich (YPD) or capsule-inducing conditions (DMEM, 37°C and 5% CO_2_). Values are normalized to the WT sample grown in YPD and are the mean ± SEM of six biological replicates.
(PDF)

S9 Fig Nucleotide sugar uptake into Gmt1- and Gmt2- containing proteoliposomes. (A) Immunoblot analysis of microsome (M) and proteoliposome (P) preparations from *S*. *cerevisiae* expressing vector only (Control) or V5-tagged Gmt1 or Gmt2 (2.5 μg protein per lane; S, molecular weight standards; C, control; 1, Gmt1; 2, Gmt2). (B and C) Representative LC-MS/MS spectra of GMP-preloaded proteoliposomes (B, Control; C, Gmt1) incubated for 10 min at 37°C with a mixture of 16 nucleotide / nucleotide sugar substrates, each 50 μM. Peak 1, GDP-Man; Peak 2, GDP-Glc; Peak 3, GDP-fucose (D and E) Quantification of nucleotide sugar uptake into proteoliposomes preloaded with (D) 30 mM GMP or (E) 30 mM UMP. Data were normalized to the total protein content of the proteoliposome preparations and show the mean ± SD of four assays. These results are consistent with prior studies [21,30] and yield new information about Gmt substrate specificity.
(PDF)

S1 Table Methylation analysis of GXM for the indicated strains. (PDF)

S2 Table Staining and stress sensitivity of *Cryptococcus neoformans* strains. (PDF)

S3 Table Nucleotide sugar contents of *Cryptococcus neoformans* strains. (PDF)

S4 Table Uxt1 and Uxt2 content of proteoliposomes used for transport assays. (PDF)
