# Supplementary material for: Xylose donor transport is critical for fungal virulence
Source: PLoS Pathog. 2018 Jan 18;14(1):e1006765. doi: 10.1371/journal.ppat.1006765 (PMC5773217; doi:10.1371/journal.ppat.1006765)
Supplement: S1 Fig — (A) Protein sequence alignment of Uxt1 and Uxt2 (CNAG_02036 and CNAG_03695) with conserved residues highlighted (black, identical residues; grey, conserved substitutions). (B) Phylogenetic relationships of C. neoformans (Cn) NSTs (including Uxt1 and Uxt2, in bold), and UDP-Xyl, UDP-Galf, and UDP-Arap transporters from other organisms (Hs, Homo sapiens; Af, Aspergillus fumigatus; At, Arabidopsis thaliana) using MUSCLE, PhyML, and TreeDyn software (see Materials and Methods). Branch lengths are drawn to scale. (PDF) [file ppat.1006765.s001.pdf]

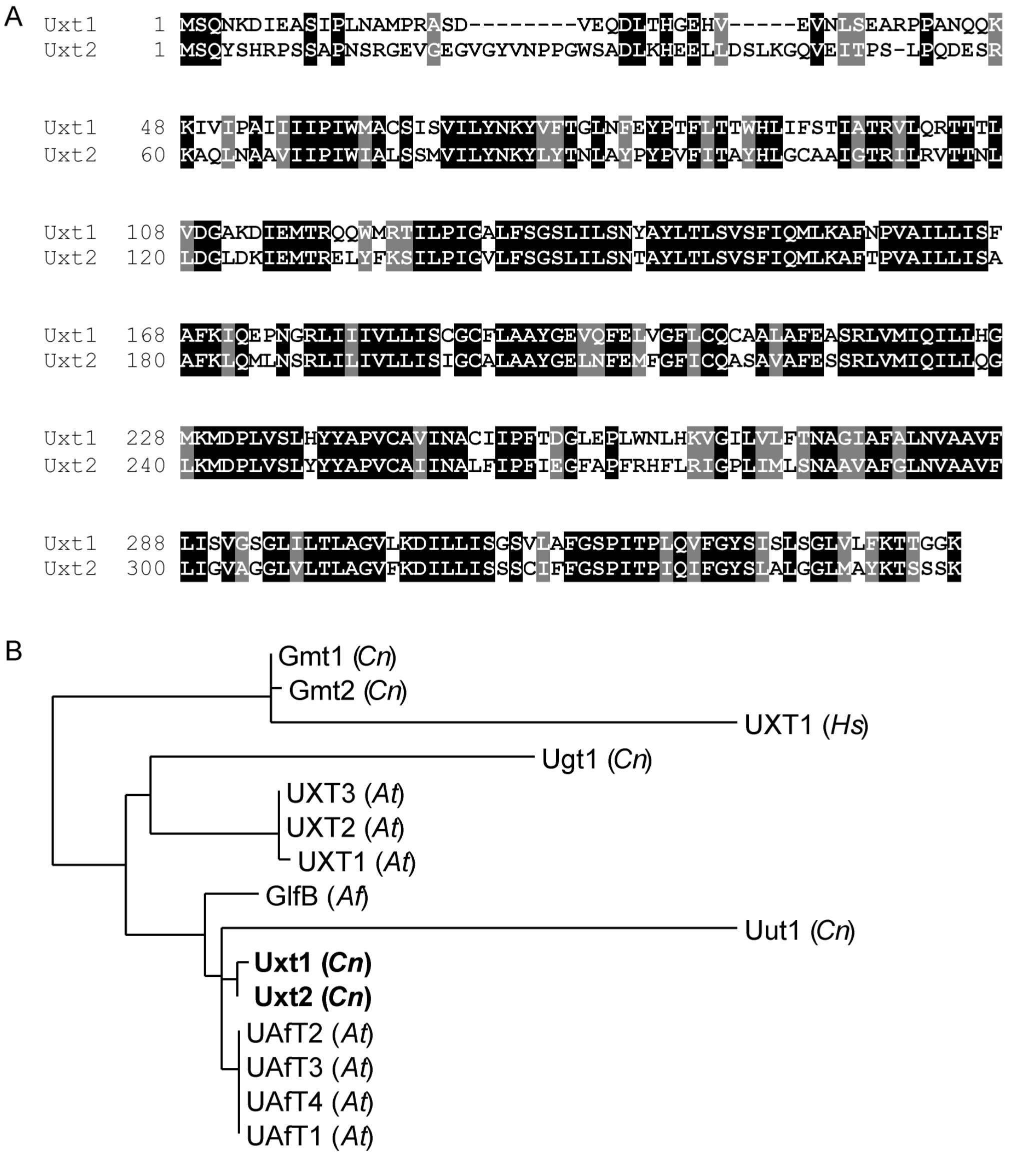

S1 Figure. Conservation of cryptococcal nucleotide sugar transporters.

(A) Protein sequence alignment of Uxt1 and Uxt2 (CNAG_02036 and CNAG_03695) with conserved residues highlighted (black, identical residues; grey, conserved substitutions). (B) Phylogenetic relationships of *C. neoformans* (*Cn*) NSTs (including Uxt1 and Uxt2, in bold), and UDP-Xyl, UDP-Gal*f*, and UDP-Ara*p* transporters from other organisms (*Hs, Homo sapiens*; *Af, Aspergillus fumigatus*; *At, Arabidopsis thaliana*) using MUSCLE, PhyML, and TreeDyn software (see Materials and Methods). Branch lengths are drawn to scale.
