# Supplementary material for: Xylose donor transport is critical for fungal virulence
Source: PLoS Pathog. 2018 Jan 18;14(1):e1006765. doi: 10.1371/journal.ppat.1006765 (PMC5773217; doi:10.1371/journal.ppat.1006765)
Supplement: S2 Fig — Cells from the indicated strains were incubated with calcofluor white (CFW; blue) to stain the cell wall and anti-GXM mAb 302 to visualize the capsule (green). Bright field, single channel, and merged images are shown; scale bar = 10 μm. cap59Δ is an acapsular strain included as a control. (PDF) [file ppat.1006765.s002.pdf]

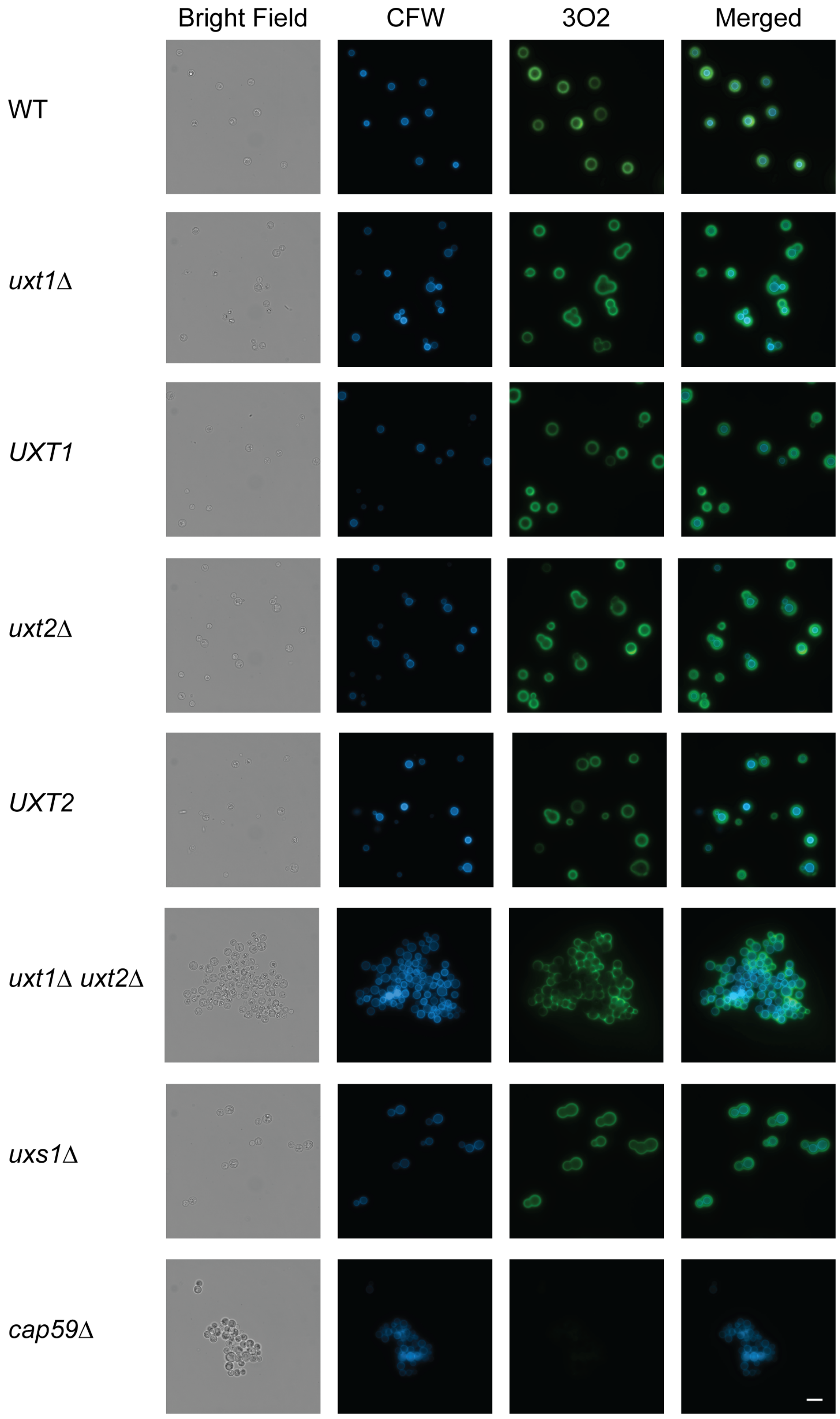

S2 Figure. *uxt1Δ uxt2Δ* is recognized by Xyl-independent capsule antibodies.

Cells from the indicated strains were incubated with calcofluor white (CFW; blue) to stain the cell wall and anti-GXM mAb 302 to visualize the capsule (green). Bright field, single channel, and merged images are shown; scale bar = 10 μm. *cap59*Δ is an acapsular strain included as a control.
