# Supplementary material for: Xylose donor transport is critical for fungal virulence
Source: PLoS Pathog. 2018 Jan 18;14(1):e1006765. doi: 10.1371/journal.ppat.1006765 (PMC5773217; doi:10.1371/journal.ppat.1006765)
Supplement: S3 Fig — (A) LC-MS/MS analysis of UDP-Galf prepared from UDP-Galp utilizing E. coli UDP-galactopyranose mutase (GLF). (B-D) Proteoliposomes prepared from S. cerevisiae expressing vector alone (B), Uxt1 (C), or Uxt2 (D) were preloaded with 30 mM UMP, and analyzed by LC-MS/MS after a 10 min incubation with 700 μM UDP-Galp and 10 μg purified GLF. Based on mass and retention time, the minor peak between UDP-Galp and UDP-Galf is likely UDP-Glc, presumably present in the reaction starting material. (E and F) Quantification of nucleotide sugar uptake into proteoliposomes preloaded with 30 mM UMP (E) or 30 mM GMP (F). Amounts were calculated using a UDP-Galp standard and normalized to the total protein content of the proteoliposome preparations and the mean ± SD of four assays are plotted. All assays were performed at 37°C. (PDF) [file ppat.1006765.s003.pdf]

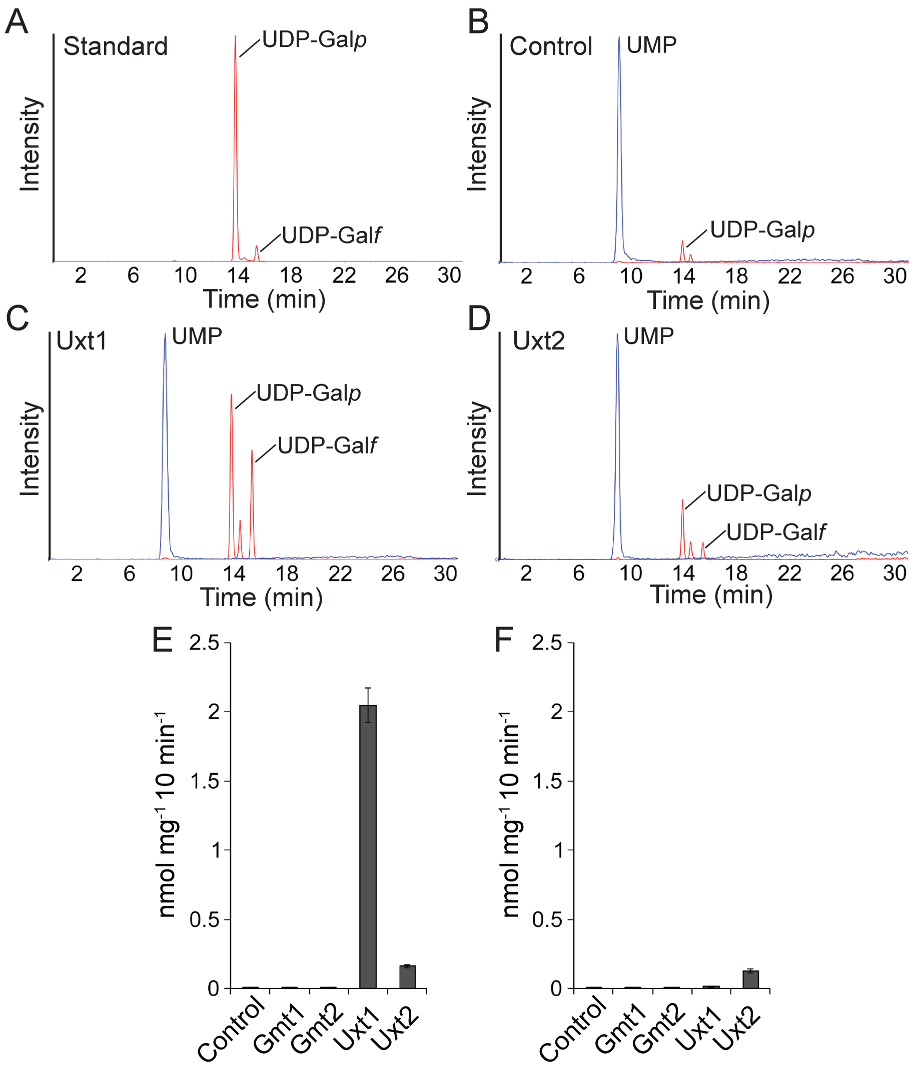

S3 Figure. Uxt1- and Uxt2-mediated UDP-Gal*f* uptake into proteoliposomes.

(A) LC-MS/MS analysis of UDP-Gal*f* prepared from UDP-Gal*p* utilizing *E. coli* UDP-galactopyranose mutase (GLF). (B - D) Proteoliposomes prepared from *S. cerevisiae* expressing vector alone (B), Uxt1 (C), or Uxt2 (D) were preloaded with 30 mM UMP, and analyzed by LC-MS/MS after a 10 min incubation with 700 μM UDP-Gal*p* and 10 μg purified GLF. Based on mass and retention time, the minor peak between UDP-Gal*p* and UDP-Gal*f* is likely UDP-Glc, presumably present in the reaction starting material. (E and F) Quantification of nucleotide sugar uptake into proteoliposomes preloaded with 30 mM UMP (E) or 30 mM GMP (F). Amounts were calculated using a UDP-Gal*p* standard

and normalized to the total protein content of the proteoliposome preparations, and the mean ± SD of four assays are plotted. All assays were performed at 37 °C.
