# Supplementary material for: Xylose donor transport is critical for fungal virulence
Source: PLoS Pathog. 2018 Jan 18;14(1):e1006765. doi: 10.1371/journal.ppat.1006765 (PMC5773217; doi:10.1371/journal.ppat.1006765)
Supplement: S4 Fig — The indicated C. neoformans strains were grown overnight at 30°C in YPD, diluted to 105 cells/mL in the media indicated, and incubated at 37°C with 5% CO2. The results shown are the averages of three measurements. Black, WT; red, uxt1Δ; green, UXT1; purple, uxt2Δ; blue, UXT2; grey, uxt1Δ uxt2Δ (continuous and dashed lines, representing three independently obtained double deletion strains). (PDF) [file ppat.1006765.s004.pdf]

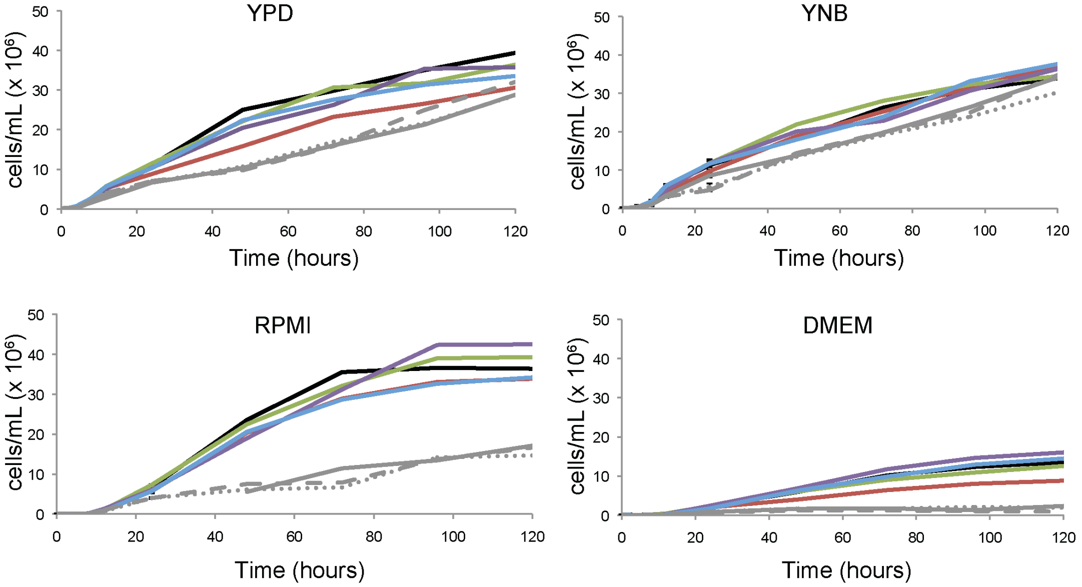

S4 Figure. *uxt1*Δ *uxt2*Δ growth is restricted at 37 °C.

The indicated *C. neoformans* strains were grown overnight at 30 °C in YPD, diluted to 10^5 cells/mL in the media indicated, and incubated at 37 °C with 5% CO_2. The results shown are the averages of three measurements. Black, WT; red, *uxt1*Δ; green, *UXT1*; purple, *uxt2*Δ; blue, *UXT2*; grey, *uxt1*Δ *uxt2*Δ (continuous and dashed lines, representing three independently obtained double deletion strains).
