# Supplementary material for: Xylose donor transport is critical for fungal virulence
Source: PLoS Pathog. 2018 Jan 18;14(1):e1006765. doi: 10.1371/journal.ppat.1006765 (PMC5773217; doi:10.1371/journal.ppat.1006765)
Supplement: S5 Fig — Induced cells were stained with India Ink, and the radius of the capsule (A) and diameter of the cell body (B) were measured using ImageJ (100 cells counted per strain; mean ± SEM of three biological replicates). (C) GXM shed from equal numbers of each of the indicated strains was quantitated by ELISA (see Materials and Methods). Data is the mean ± SEM of three independent experiments. *, p < 0.05, one-way ANOVA with Tukey’s post-hoc test. (D) Electron micrographs of the indicated strains induced for capsule as in Fig 5. Two representative images are displayed for each strain. Scale bar = 0.5 μm. (PDF) [file ppat.1006765.s005.pdf]

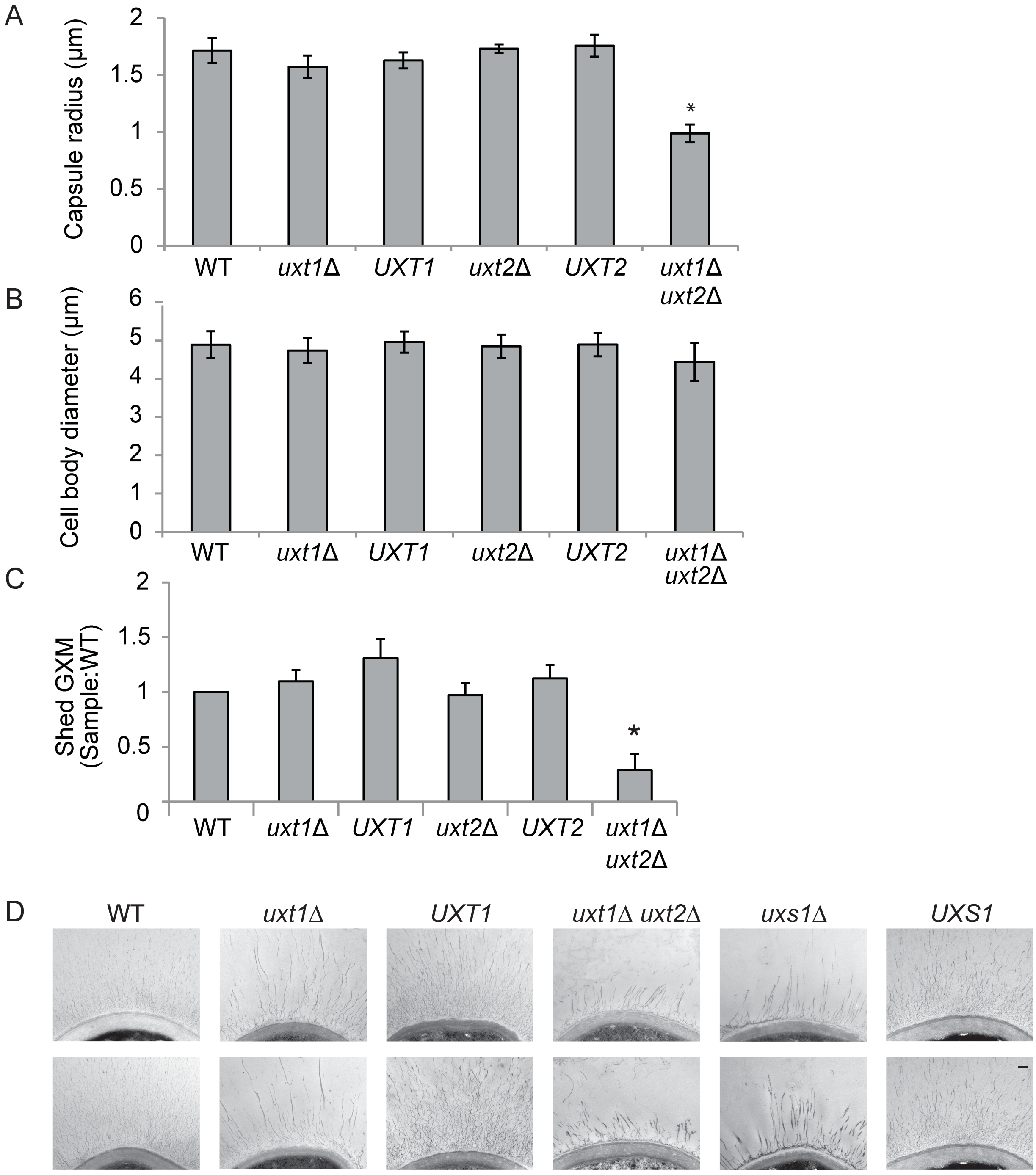

S5 Figure. Morphological defects of *uxt1Δ uxt2Δ*.

Induced cells were stained with India Ink, and the radius of the capsule (A) and diameter of the cell body (B) were measured using ImageJ (100 cells counted per strain; mean ± SEM of three biological replicates). (C) GXM shed from equal numbers

of each of the indicated strains was quantitated by ELISA (see Materials and Methods). Data is the mean ± SEM of three independent experiments. *, $p < 0.05$, one-way ANOVA with Tukey's *post-hoc* test. (D) Electron micrographs of the indicated strains induced for capsule as in Fig. 5. Two representative images are displayed for each strain. Scale bar = 0.5 μm.
