# Supplementary material for: Xylose donor transport is critical for fungal virulence
Source: PLoS Pathog. 2018 Jan 18;14(1):e1006765. doi: 10.1371/journal.ppat.1006765 (PMC5773217; doi:10.1371/journal.ppat.1006765)
Supplement: S6 Fig — Survival of C57BL/6 mice after intranasal inoculation with 5 × 10^4^ cells of WT (n = 5) or uxt1Δ uxt2Δ (n = 19). C57BL/6 mice naturally skew towards a non-protective Th2-type response, which increases their susceptibility to cryptococcal infection compared to A/JCr mice [57]. (PDF) [file ppat.1006765.s006.pdf]

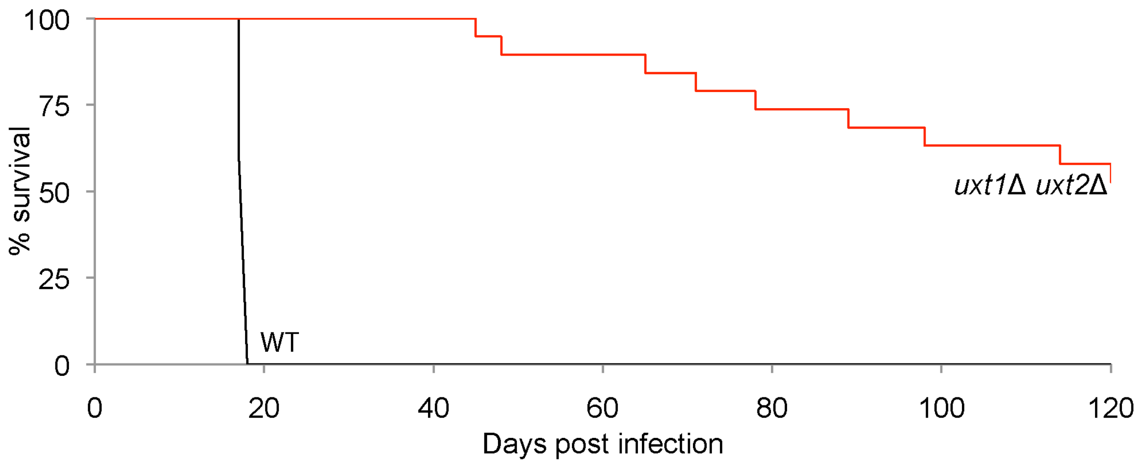

S6 Figure. *uxt1*Δ *uxt2*Δ is severely attenuated for virulence in C57BL/6 mice. Survival of C57BL/6 mice after intranasal inoculation with 5×10^4 cells of WT (n = 5) or *uxt1*Δ *uxt2*Δ (n = 19). C57BL/6 mice naturally skew towards a non-protective Th2-type response, which increases their susceptibility to cryptococcal infection compared to A/JCr mice [57].
