# Supplementary material for: Xylose donor transport is critical for fungal virulence
Source: PLoS Pathog. 2018 Jan 18;14(1):e1006765. doi: 10.1371/journal.ppat.1006765 (PMC5773217; doi:10.1371/journal.ppat.1006765)
Supplement: S7 Fig — Brain (A) and spleen (B) CFU of infected A/JCr mice at the time of death (for WT, uxt1Δ, uxt2Δ, and complemented mutants; n = 8) or at the indicated time points (for uxt1Δ uxt2Δ; n = 3). Open circles, individual mice; black bar, mean; dashed line, initial inoculum. **, p < 0.01 by one-way ANOVA with Tukey’s post hoc test. (PDF) [file ppat.1006765.s007.pdf]

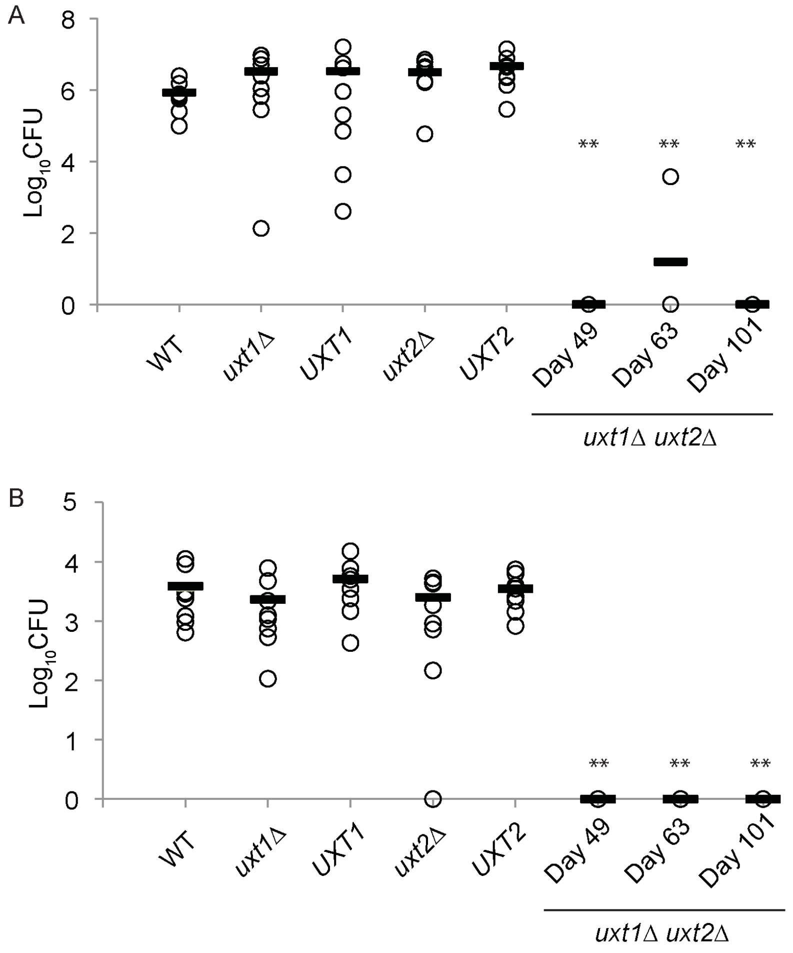


**S7 Figure. *uxt1Δ uxt2Δ* does not colonize extrapulmonary sites.**

Brain (A) and spleen (B) CFUs of infected A/JCr mice at the time of death (for WT, *uxt1*Δ, *uxt2*Δ, and complemented mutants; n = 8) or at the indicated time points (for *uxt1*Δ *uxt2*Δ; n = 3). Open circles, individual mice; black bar, mean; dashed line, initial inoculum. **, $p < 0.01$ by one-way ANOVA with Tukey's post hoc test.
