# Supplementary material for: Xylose donor transport is critical for fungal virulence
Source: PLoS Pathog. 2018 Jan 18;14(1):e1006765. doi: 10.1371/journal.ppat.1006765 (PMC5773217; doi:10.1371/journal.ppat.1006765)
Supplement: S8 Fig — Expression of UXT1 and UXT2 measured by qRT-PCR with RNA prepared from the indicated strains after growth in nutrient rich (YPD) or capsule-inducing conditions (DMEM, 37°C and 5% CO2). Values are normalized to the WT sample grown in YPD and are the mean ± SEM of six biological replicates. (PDF) [file ppat.1006765.s008.pdf]

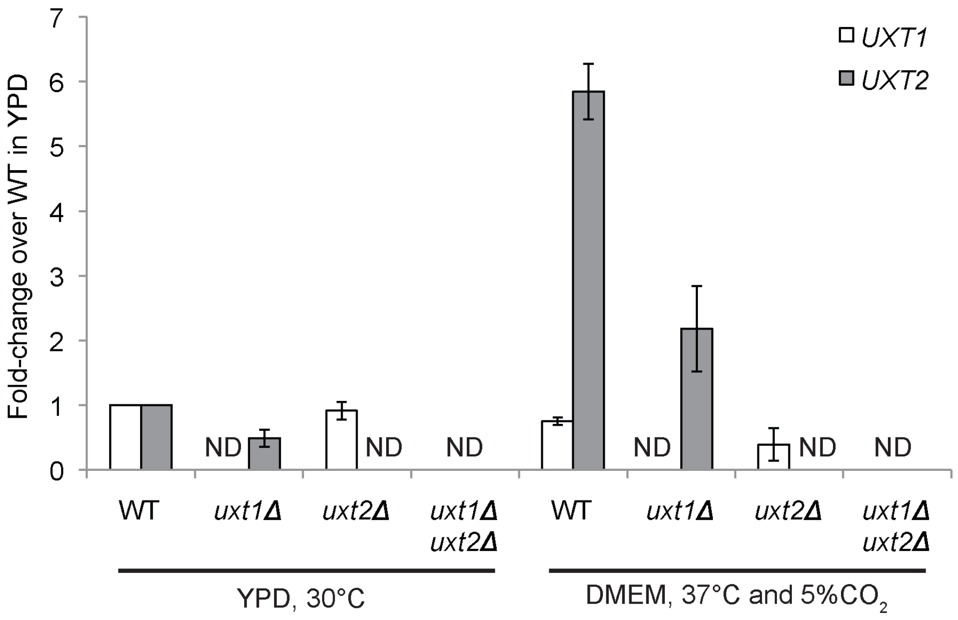

S8 Figure. *UXT1* and *UXT2* transcription levels.

Expression of *UXT1* and *UXT2* measured by qRT-PCR with RNA prepared from the indicated strains after growth in nutrient rich (YPD) or capsule-inducing conditions (DMEM, 37 °C and 5% CO_2). Values are normalized to the WT sample grown in YPD and are the mean ± SEM of six biological replicates.
