# Supplementary material for: Xylose donor transport is critical for fungal virulence
Source: PLoS Pathog. 2018 Jan 18;14(1):e1006765. doi: 10.1371/journal.ppat.1006765 (PMC5773217; doi:10.1371/journal.ppat.1006765)
Supplement: S9 Fig — (A) Immunoblot analysis of microsome (M) and proteoliposome (P) preparations from S. cerevisiae expressing vector only (Control) or V5-tagged Gmt1 or Gmt2 (2.5 μg protein per lane; S, molecular weight standards; C, control; 1, Gmt1; 2, Gmt2). (B and C) Representative LC-MS/MS spectra of GMP-preloaded proteoliposomes (B, Control; C, Gmt1) incubated for 10 min at 37°C with a mixture of 16 nucleotide / nucleotide sugar substrates, each 50 μM. Peak 1, GDP-Man; Peak 2, GDP-Glc; Peak 3, GDP-fucose (D and E) Quantification of nucleotide sugar uptake into proteoliposomes preloaded with (D) 30 mM GMP or (E) 30 mM UMP. Data were normalized to the total protein content of the proteoliposome preparations and show the mean ± SD of four assays. These results are consistent with prior studies [21,30] and yield new information about Gmt substrate specificity. (PDF) [file ppat.1006765.s009.pdf]

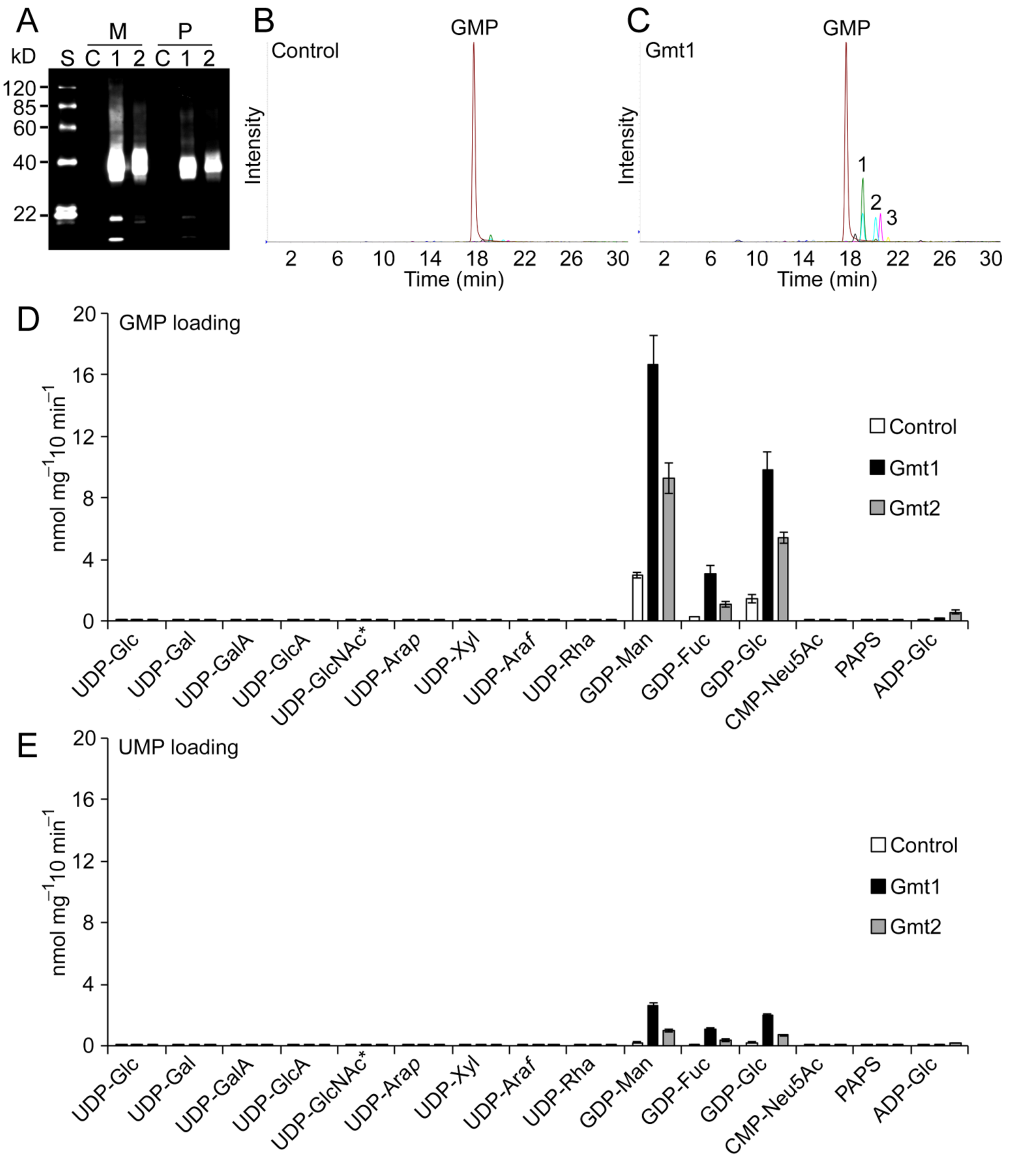


**S9 Figure. Nucleotide sugar uptake into Gmt1- and Gmt2- containing proteoliposomes.**

(A) Immunoblot analysis of microsome (M) and proteoliposome (P) preparations from *S. cerevisiae* expressing vector only (Control) or V5-tagged Gmt1 or Gmt2 (2.5 µg protein per lane; S, molecular weight standards; C, control; 1, Gmt1; 2, Gmt2). (B and C) Representative LC-MS/MS spectra of GMP-preloaded proteoliposomes (B, Control; C, Gmt1) incubated for 10 min at 37 °C with a mixture of 16 nucleotide / nucleotide sugar substrates, each 50 mM. Peak 1, GDP-Man; Peak 2, GDP-Glc; Peak 3, GDP-fucose (D

and E) Quantification of nucleotide sugar uptake into proteoliposomes preloaded with (D) 30 mM GMP or (E) 30 mM UMP. Data were normalized to the total protein content of the proteoliposome preparations and show the mean ± SD of four assays. These results are consistent with prior studies [21, 30] and yield new information about Gmt substrate specificity.
