# Supplementary material for: Xylose donor transport is critical for fungal virulence
Source: PLoS Pathog. 2018 Jan 18;14(1):e1006765. doi: 10.1371/journal.ppat.1006765 (PMC5773217; doi:10.1371/journal.ppat.1006765)
Supplement: S1 Table — (PDF) [file ppat.1006765.s010.pdf]

S1 Table. Methylation analysis of GXM for the indicated strains.[a]

	WT	*uxt1Δ*	*UXT1*	*uxt2Δ*	*UXT2*	*uxt1Δ uxt2Δ*
t-Man	0.4	0.1	0.1	0.3	0.1	0.5
3-Man	42	66	47	51	39	80[b]
2,3-Man	53	33	48	45	54	18
3,4-Man	0.8	0.4	0.6	0.8	1.4	0.8
2,3,4-Man	3.4	0.4	4.5	2.5	4.1	0.0

[a] Values reported as percent of total mannose linkages to facilitate comparison.

[b] This product primarily reflects backbone substitution with GlcA.
