# Supplementary material for: Xylose donor transport is critical for fungal virulence
Source: PLoS Pathog. 2018 Jan 18;14(1):e1006765. doi: 10.1371/journal.ppat.1006765 (PMC5773217; doi:10.1371/journal.ppat.1006765)
Supplement: S2 Table — (PDF) [file ppat.1006765.s011.pdf]

S2 Table. Staining and stress sensitivity of *Cryptococcus neoformans* strains.

	Anti-GXM mAbs		Cell wall staining[a]				Cell growth[b]				
Strain	2H1	3C2	CFW	ConA	EosinY	Pont	YPD	2% CFW	0.05% CR	0.01% SDS	1.5 M Sorbitol
WT	+	+	+	+	+	+	+	+	+	+	+
*uxt1*Δ	+	+	+	+	+	+	+	+	+	+	+
UXT1	+	+	+	+	+	+	+	+	+	+	+
*uxt2*Δ	+	+	+	+	+	+	+	+	+	+	+
UXT2	+	+	+	+	+	+	+	+	+	+	+
*uxt1*Δ *uxt2*Δ	-	-	+	+	+	+	+	+	+	+	+
*uxs1*Δ	-	-	NT[c]	NT	NT	NT	+	+	+	+	+

[a] The indicated strains were stained with CFW (binds chitin), ConA (binds mannoproteins), Eosin Y (binds chitosan), or Pont (binds unspecified cell wall components) as in Materials and Methods.

[b]All growth conditions were assayed at 30°C.

[c]NT, not tested
