# Supplementary material for: Xylose donor transport is critical for fungal virulence
Source: PLoS Pathog. 2018 Jan 18;14(1):e1006765. doi: 10.1371/journal.ppat.1006765 (PMC5773217; doi:10.1371/journal.ppat.1006765)
Supplement: S3 Table — (PDF) [file ppat.1006765.s012.pdf]

S3 Table**.** Nucleotide sugar contents of *Cryptococcus neoformans* strains.[a]

Compound	Wild type	*uxt1*Δ	*UXT1*	*uxt2*Δ	*UXT2*	*uxt1*Δ *uxt2*Δ	*uxs1*Δ
UDP-α-D-Xyl	1 ± 0[b]	3 ± 1	2 ± 1	1 ± 0	1 ± 0	6 ± 3	ND[c,d]
UDP-α-D-Glc	16 ± 2	55 ± 17	26 ± 7	23 ± 6	21 ± 6	46 ± 21	50 ± 9
UDP-α-D-GlcA	4 ± 1	4 ± 2	5 ± 1	6 ± 1	5 ± 2	0 ± 0	400 ± 105[c]
UDP-α-D-Gal*p*	1 ± 0	4 ± 3	2 ± 1	1 ± 1	1 ± 1	3 ± 3	4 ± 3

[a] Levels of UDP-α-D-Gal*f*, UDP-α-D-Ara*p*, and UDP-α-D-Ara*f* were below the limit of detection.

[b] Values are given in pmol mg^{-1} wet weight and represent the average of $n = 4$ (± SEM). Estimated cell volume was used to convert values to μM (see Materials and Methods for details).

[c] ANOVA, $p \leq 0.01$.

[d] ND, below limit of detection.
