# Supplementary material for: Xylose donor transport is critical for fungal virulence
Source: PLoS Pathog. 2018 Jan 18;14(1):e1006765. doi: 10.1371/journal.ppat.1006765 (PMC5773217; doi:10.1371/journal.ppat.1006765)
Supplement: S4 Table — (PDF) [file ppat.1006765.s013.pdf]

S4 Table. Uxt1 and Uxt2 content of proteoliposomes used for transport assays.

	Molecular Mass (Da)	fmol[a]/5 µg	ng/5 µg	Total protein (%)
Uxt1	42442.2	501.8 ± 2.0[b]	21.3 ± 0.1[b]	0.43 ± 0.00[b]
Uxt2	43535.4	116.2 ± 5.9[b]	5.1 ± 0.3[b]	0.10 ± 0.01[b]

[a]Amount was estimated using LC-MS/MS (MRM) quantitation of a C-terminal peptide (SRGPFEGKPIPNPLLGLDSTR), and interpreted based on the molecular mass (including V5-tag and 6-His tags) estimated using the Compute pI/Mw tool at ExPASy (http://web.expasy.org/).

[b]Values represent the mean ± SD of n = 3.
